# Konsensusempfehlungen zur Diagnose und Therapie der Hyponatriämie der Österreichischen Gesellschaft für Nephrologie 2024

**DOI:** 10.1007/s00508-024-02325-5

**Published:** 2024-02-29

**Authors:** Christoph Schwarz, Gregor Lindner, Martin Windpessl, Maarten Knechtelsdorfer, Marcus D. Saemann

**Affiliations:** 1 Innere Medizin 1, Pyhrn-Eisenwurzenklinikum, Sierningerstr. 170, 4400 Steyr, Österreich; 2 Zentrale Notaufnahme, Kepler Universitätsklinikum GmbH, Johannes-Kepler-Universität, Linz, Österreich; 3 Innere Medizin IV, Klinikum Wels-Grieskirchen, Wels, Österreich; 4 6.Medizinische Abteilung mit Nephrologie und Dialyse, Klinik Ottakring, Wien, Österreich; 5 Medizinische Fakultät, Sigmund-Freud Universität, Wien, Österreich

**Keywords:** Osmotisches Demyelinisierungssyndrom, Polydipsie, Osmolalität, Antidiuretisches Hormon, Hirnödem, Osmotic demyelination syndrome, Polydipsia, Osmolality, Vasopressin, Cerebral edema

## Abstract

Die Hyponatriämie ist eine Störung des Wasserhaushaltes. Die Wasserhomöostase wird durch das Zusammenspiel von Nierenfunktion und den zerebralen Strukturen des Durstempfindens und der Produktion des antidiuretischen Hormons aufrechterhalten. Durch die Messung der Serum-Osmolalität, Harn-Osmolalität und Harn- Natriumkonzentration können die meisten Ursachen der Hyponatriämie identifiziert werden. Hyponatriämien führen zu einem Hirnödem und können damit schwere neurologische Symptome verursachen, welche eine akute Therapie benötigen. Aber auch milde Formen der Hyponatriämie bedürfen einer, wenn möglich kausalen, oder zumindest symptomatischen Behandlung. Eine inadäquat rasche Korrektur der Hyponatriämie sollte vermieden werden, da diese das Risiko für ein zerebrale osmotische Demyelinisierung erhöht. Da die Art der Therapie eng mit der Ursache der Hyponatriämie zusammenhängt, ist eine Grundkenntnis der pathophysiologischen Prozesse für eine optimale Behandlung notwendig.

## Abstract (en)

Hyponatremia is a disorder of water homeostasis. Water balance is maintained by the collaboration of renal function and cerebral structures, which regulate thirst mechanisms and secretion of the antidiuretic hormone. Measurement of serum-osmolality, urine osmolality and urine-sodium concentration help to diagnose the different reasons for hyponatremia. Hyponatremia induces cerebral edema and might lead to severe neurological symptoms, which need acute therapy. Also, mild forms of hyponatremia should be treated causally, or at least symptomatically. An inadequate fast increase of the serum sodium level should be avoided, because it raises the risk of cerebral osmotic demyelination. Basic pathophysiological knowledge is necessary to identify the different reasons for hyponatremia which need different therapeutic procedures.

## Einleitung

Die Hyponatriämie hat eine hohe Prävalenz, ist klinisch relevant, betrifft nahezu alle Bereiche der Medizin und ist ein Symptom von verschiedenen Erkrankungen. Die mit ihr assoziierte erhöhte Morbidität und Mortalität sind zum einen durch die zugrunde liegende Ätiologie bedingt, andererseits weist die Hyponatriämie selbst einen Krankheitswert auf, da sie zu Symptomen wie Schwindel, erhöhter Sturzneigung bis hin zum Koma führen kann. Auch eine inadäquate Therapie birgt Gefahren wie ein osmotisches Demyelinisierungssyndrom (ODS). Deshalb ist es für jede:n Arzt:Ärztin essenziell, die Grundlagen zur Entstehung, Differentialdiagnose und Therapie der Hyponatriämie zu kennen.

## Definition der Hyponatriämie

Eine Hyponatriämie ist definiert durch eine Verminderung des Serum-Natriums (S-Na^+^) unterhalb des unteren Referenzbereichs des ausführenden Labors. Dies entspricht in der Regel einem S‑Na^+^ < 135 mmol/l [1].

### A) Klassifikation der Hyponatriämie

#### a) Biochemische Einteilung

- **mild:** S‑Na^+^ 130–134 mmol/l
- **moderat:** S‑Na^+^ 125–129 mmol/l
- **profund**: S‑Na^+^ < 125 mmol/l [1]

#### b) Klinischer Schweregrad (Symptomatik)

Der Schweregrad der klinischen Symptomatik korreliert nicht immer mit dem biochemischen Ausmaß der Hyponatriämie. Die Klinik ist abhängig vom zeitlichen Rahmen der Entwicklung der Hyponatriämie [2]. Ein rascher Abfall des S‑Na^+^ führt zu einer stärkeren klinischen Symptomatik, bedingt durch eine ausgeprägtere Entwicklung eines Hirnödems [3]. Ebenso ist die klinische Symptomatik in der Regel stärker bei jüngeren Patient:innen ausgeprägt. Die klinischen Symptome sind nicht pathognomonisch für eine Hyponatriämie (Tab. 1).

**Tab. 1:** Schweregrad der klinischen Symptomatik

*Leicht*	Es bestehen keine klinisch fassbaren Symptome, aber dennoch Einschränkungen bestimmter zerebraler Funktionen (z. B. Aufmerksamkeit), welche nur durch spezifische kognitive Tests erhoben werden können [4]. Das Risiko für Stürze und Frakturen ist erhöht [4, 5]
*Mittel* [1]	Übelkeit, neu aufgetretene Verwirrtheit, Kopfschmerzen, Schwindel, Muskelkrämpfe
*Schwer* [1]	= potenziell lebensbedrohliche Symptome Somnolenz, zerebraler Krampfanfall, hämodynamische Zeichen eines erhöhten Hirndrucks (Blutdruckanstieg, Bradykardie, niedrige Atemfrequenz), Übelkeit mit Erbrechen (nur in Kombination mit weiteren schweren Symptomen)

#### c) Zeitlicher Verlauf

Akut (< 48h): Es kann nachgewiesen werden, dass der letzte gemessene S‑Na^+^-Wert innerhalb von 48 h > 135 mmol/l war [1].

Chronisch (> 48 h): jede Hyponatriämie, die vor mehr als 48 h erstmals nachweisbar war. Wenn kein S‑Na^+^ > 135 mmol/l innerhalb der letzten 48 h vorliegt, sollte die Hyponatriämie als chronisch klassifiziert werden [1].

## Epidemiologie

Hyponatriämien sind selbst in der Allgemeinbevölkerung außerhalb des Krankenhauses mit einer Prävalenz von bis zu 5 % der Erwachsenen häufig [6]. Die Prävalenz steigt deutlich auf über 20 % bei älteren Patient:innen (> 65 Jahre) und auf bis zu 35 % bei hospitalisierten Menschen [6–8]. Patient:innen mit Herzinsuffizienz (bis zu 30 %), mit Tumor oder Lebererkrankung (bis zu 50 %) sind besonders häufig davon betroffen [9]. Profunde Hyponatriämien < 125 mmol/l sind hingegen insgesamt deutlich seltener (ca. 2 %) [10].

Bislang existieren kaum Studien, welche spezifisch die Implikationen des biologischen Geschlechts auf die Inzidenz und die Auswirkungen einer Hyponatriämie untersuchten. Nur in einer großen Kohorte zeigte sich männliches Geschlecht als ein diskreter Prädiktor für das Vorliegen einer Hyponatriämie [6]. In anderen retrospektiven Analysen war hingegen das weibliche Geschlecht mit einer erhöhten Hyponatriämieprävalenz assoziiert [11, 12]. Mit steigendem Alter nimmt das Vorliegen einer Hyponatriämie bei Frauen im Vergleich zu Männern überproportional zu [11]. Bei speziellen Ätiologien wie der „excercise-associated hyponatremia“ oder der Thiazid-assoziierten Hyponatriämie ist die Prävalenz bei Frauen ebenfalls höher im Vergleich zu Männern [13, 14].

Chronische Hyponatriämien sind mit langen Krankenhausaufenthalten, erhöhten Kosten des Gesundheitssystems und auch mit erhöhter Mortalität assoziiert [15, 16]. Dabei ist nicht immer eindeutig, ob die Hyponatriämie ein kausaler Faktor für die Mortalität ist oder vielmehr ein Zeichen für den Schweregrad einer Erkrankung, welche zu einer Hyponatriämie führt [17]. Eine Hyponatriämie ist ein Risikofaktor für eine erhöhte Mortalität bei verschiedenen Erkrankungen (z. B. Pneumonie, Herzinsuffizienz, Nierenkrankheit, kritisch kranken Patient:innen) [18–21]. Insbesondere akute Hyponatriämien sind unbehandelt mit einer hohen Mortalität vergesellschaftet (bis zu 50 %) [22]. Weibliches Geschlecht war zumindest in einer gepoolten Studie mit mehr als 14.000 Patient:innen mit der ICD-10 Diagnose Hyponatriämie oder SIAD mit einer deutlich geringeren Letalität assoziiert (OR 0,56, 0,49–0,64) [23].

Niedrige Serum-Natriumkonzentrationen sind auch mit einer erhöhten Morbidität assoziiert [24]. Sie führen zu vermehrten Stürzen, Osteoporose und Frakturen, weshalb sie auch eine erhebliche finanzielle Belastung für das Gesundheitssystem darstellen [25–27].

## Physiologie des Wasserhaushaltes

### A) Wie entsteht eine Hyponatriämie?

Eine Hyponatriämie ist prinzipiell eine *Störung des Wasserhaushaltes*. Die Serum-Natriumkonzentration wird durch die Gesamtmenge an austauschbarem Natrium und Kalium sowie dem gesamten Körperwasser bestimmt, wie aus der vereinfachten Edelman-Gleichung ersichtlich ist [28].

1 $$\left[\mathrm{Na}^{+}\right]=\frac{\left(\mathrm{Na}\right)e+\left(\mathrm{K}\right)e}{\left(\mathrm{TBW}\right)}$$

Na(e)…: Gesamtkörper Natrium („exchangeable“; frei austauschbar) in mmol,
K(e)…: Gesamtkörper Kalium („exchangeable“; frei austauschbar) in mmol,
TBW…: Gesamtkörper Wasser in l.

Es ist wichtig zu beachten, dass es im Körper osmotisch inaktive, nicht austauschbare Natriumspeicher gibt (in der Haut, im Knorpelgewebe und im Knochen), die nicht in die Formel mit einbezogen werden [29]. Kalium ist in der Formel enthalten, da die Edelman-Gleichung gleiche Konzentrationen gelöster Stoffe über die Zellmembranen impliziert, wobei Natrium das wichtigste extra- und Kalium das wichtigste intrazelluläre Ion ist. Da Wasser die Zellwand passieren kann, ist die Konzentration gelöster Stoffe intra- und extrazellulär gleich.

Nach der Edelman-Gleichung kann sich eine Hyponatriämie nur entwickeln, wenn entweder ein Natriumverlust, ein Kaliumverlust, ein Wassergewinn oder eine Kombination aus diesen Faktoren vorliegt [28]. Daraus kann geschlossen werden, dass auch ein Anstieg des gesamten austauschbaren Kaliums die Natriumkonzentration im Serum beeinflussen kann. Dies ist für das Management einer Hyponatriämie wichtig, wenn ein Patient:in sowohl mit Hyponatriämie als auch mit Hypokaliämie behandelt wird: Die Substitution von Kalium kann deshalb zu einem schnelleren Anstieg des S‑Na^+^ führen [30].

Darüber hinaus kann sich eine Hyponatriämie durch eine Verlagerung von freiem Wasser vom intrazellulären in den extrazellulären Raum entwickeln (translokationale Hyponatriämie), verursacht durch eine osmotisch aktive Substanz, die die Zellmembran nicht ungehindert passieren kann. Eine Hyperglykämie ist die wichtigste Ursache dieses Phänomens.

**Abb. 1:**
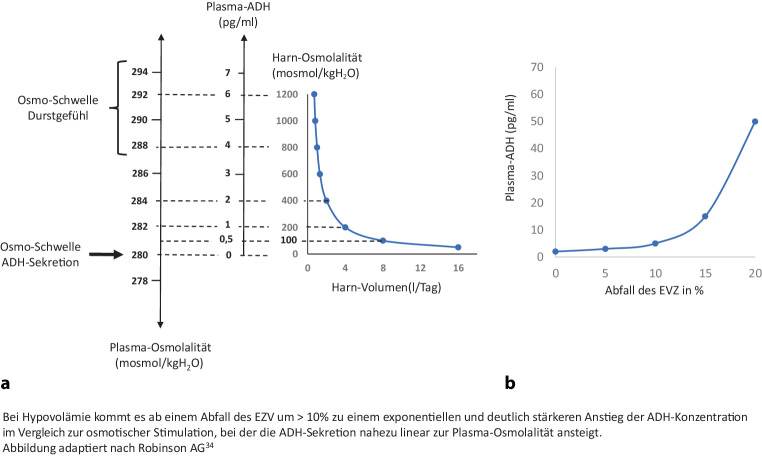
ADH-Sekretion in Relation zur Plasmaosmolalität und Extrazellularvolumen. **a** Plasma-ADH, Harn/Plasmaosmolalität und Durst. **b** Plasma-ADH bei Änderung des Extrazellularvolumens und konstanter Plasmaosmolalität

Wie durch die Edelman-Gleichung ausgedrückt, ist die Serum-Natriumkonzentration ein Ergebnis des Verhältnisses der Natriummenge und des Gesamtkörperwassers [28]. Daher spiegelt die Serum-Natriumkonzentration den Zustand der Wasserhomöostase wider. Die Entwicklung von Durst, Trinken, Durstsättigung und die Wirkung des antidiuretischen („wassersparenden“) Hormons (ADH) sind für die Regulierung des Körperwassers von wesentlicher Bedeutung [31]. Wenn die Plasmaosmolalität auf über ca. 280 mOsmol/kgH_2_O ansteigt, wird ADH ausgeschüttet und bei etwas höheren Werten entsteht zusätzlich ein Durstgefühl [32, 33]. Folglich wird Flüssigkeit getrunken und durch Vermittlung von ADH im Sammelkanal der Niere (mittels Aquaporinen) freies Wasser aus dem Urin rückresorbiert. Dadurch erreicht die Plasmaosmolalität wieder den Normalbereich (s. Abb. 1; [34]). Bemerkenswert ist, dass die ADH-Sekretion auch durch dehnungsempfindliche Barorezeptoren (lokalisiert u. a. im Aortenbogen und den Karotiden) reguliert wird: Bereits ein geringes Absinken des Blutdrucks oder des Extrazellularvolumens führt zu einer erhöhten ADH-Sekretion (s. Abb. 1; [34, 35]). Bei schwererer Hypovolämie mit reduziertem effektivem arteriellem Blutvolumen (EABV) kann die Baroregulation die Osmoregulation außer Kraft setzen, sodass es trotz ausgeprägter Hypoosmolalität zu einer ADH-Sekretion kommt („*Baroregulation kommt vor Osmoregulation*“) [36].

### B) Altersspezifische physiologische Veränderungen

Mit zunehmendem Alter nimmt die Kapazität ab, die Wasserhomöostase bei interkurrenten Erkrankungen oder äußeren Faktoren (z. B. Hitze, sehr große Trinkmengen) aufrechtzuerhalten. Die Fähigkeit der Niere, Wasser zu eliminieren, sinkt durch den physiologischen altersbedingten Abfall der glomerulären Filtrationsrate (GFR) und durch eine Beeinträchtigung der maximalen Harndilutionskapazität, sodass trotz fehlender ADH-Sekretion die Harnosmolalität bis 150 mOsmol/kgH_2_O liegen kann. Für die höhere Prävalenz eines SIAD im Alter spielt eine höhere basale ADH-Sekretion bzw. eine geringere Suppression der ADH-Sekretion nach dem Trinken eine wesentliche Rolle [37]. Zusätzlich sind ein geringeres Gesamtkörperwasser und eine Änderung der Ernährungsgewohnheiten („low solute intake“) Risikofaktoren für die Entwicklung einer Hyponatriämie. Schließlich leiden ältere Menschen häufiger an Erkrankungen, welche mit einer Hyponatriämie assoziiert sind, oder nehmen öfter Medikamente ein, welche die Harndilution beeinträchtigen [38].

### C) Geschlechtsspezifische physiologische Unterschiede

Mögliche Gründe für das höhere Risiko von Frauen, eine Hyponatriämie zu entwickeln, sind eine andere Zusammensetzung des Gesamtkörperwassers (♂ ca. 60 %, ♀ ca. 50 %) bei gleichzeitig oft niedrigerem Body-Mass-Index sowie auch eine erhöhte ADH-Sensitivität [39]. Zusätzlich kommt es während des Menstruationszyklus zu einer Änderung der Konzentrationen von Progesteron und Östrogen. Während Progesteron v. a. die renale Natriumausscheidung und den Salzhunger beeinflusst, bewirkt Östrogen eine Änderung der Durstschwelle und der ADH-Sekretion hin zu einer niedrigeren S‑Osmolalität [40]. Der Grund für eine höhere Prävalenz einer „excercise associated hyponatremia“ bei Frauen begründet sich u. a. auch durch eine höhere Flüssigkeitszufuhr [13]. Die physiologischen Veränderungen während der Schwangerschaft sind weiter unten beschrieben.

## Diagnostische Parameter

### A) Anamnese

Die Anamnese dient dazu, klinische Risikofaktoren für die Entwicklung einer Hyponatriämie zu erkennen. Die Erhebung der Trinkmenge (= Zufuhr von Wasser ohne Elektrolyten), der Nahrungszufuhr (v. a. Salz und Eiweiß = osmotische Substanzen) und Situationen, die zu Verlusten an Elektrolyten und Wasser (z. B. Diarrhö, Fieber) führen, sind dabei wichtige Parameter. Eine sorgfältige Medikamentenanamnese mit Augenmerk auf Hyponatriämie-induzierende Wirkstoffe wie Diuretika ist zentral. Besonderes Augenmerk sollte auf Antihypertensiva gelegt werden, da diese häufig als Komponente ein Thiaziddiuretikum enthalten, welches das Auftreten einer Hyponatriämie verursachen kann [14, 41].

### B) Labor

#### a) Serum-Natrium (mmol/l)

Aufgrund der raschen Verfügbarkeit und kurzen Analysezeit kann bei Verdacht auf eine Hyponatriämie primär eine Messung mit einem Blutgasanalysegerät erfolgen. Im weiteren Verlauf sollten aufgrund der geringeren Variabilität der Messwerte die Messung der S‑Natriumkonzentration aus dem venösen Blut und die Analyse im Zentrallabor bevorzugt werden. Die Messwerte aus der Blutgasanalyse können nicht direkt mit jenen aus dem Zentrallabor verglichen werden [42]. Im klinischen Alltag wird eine Hyponatriämie aber zumeist im Rahmen der routinemäßigen Blutabnahme zum Zeitpunkt der Aufnahme oder während des stationären Aufenthaltes erkannt.

Durch Messung der Natriumkonzentration mit einer Blutgasanalyse (direkte Na^+^-Messung mit ionenselektiver Elektrode) kann eine Pseudohyponatriämie ausgeschlossen werden (für Details s. Abschnitt „Nichthypotone Hyponatriämien“).

#### b) Serumosmolalität-gemessen (mOsmol/kgH_2_O)

In die Messung der S‑Osmolalität gehen *alle* osmotisch wirksamen Bestandteile des Serums ein [43]. Es werden sowohl osmotisch effektive Substanzen (Natrium, Kalium, Glukose, organische Osmolyte), welche durch ihre eingeschränkte Permeabilität durch die Zellwand einen osmotischen Gradienten aufbauen können, als auch osmotisch ineffektive Substanzen (Ethanol, Harnstoff), welche frei permeabel sind und deshalb keine osmotischen Gradienten aufbauen können, gemessen. Relevant für eine klinische Symptomatik ist jedoch nur die Gesamtkonzentration der effektiven osmotischen Substanzen, da nur diese einen transzellulären Shift von Wasser auslösen können [10]. Die Tonizität im Serum wird ausschließlich durch diese effektiv osmotischen Substanzen definiert [44]. Die Berechnung der S‑Osmolalität hat Limitationen und kann nicht empfohlen werden; sie ist aber eine wertvolle Ergänzung in der Abklärung einer iso-hypertonen Hyponatriämie (s. Supplement 2). Alternativ kann auch die Osmolalität im Plasma gemessen werden, die Referenzwerte sind ident zur S‑Osmolalität [45].

Eine niedrige gemessene S‑Osmolalität diagnostiziert eine hypotone („echte“) Hyponatriämie. Nur diese verursacht ein Hirnödem mit daraus folgender möglicher klinischer Symptomatik.

#### c) Harnosmolalität (mOsmol/kgH_2_O) gemessen oder berechnet

Die Harnosmolalität ist ein indirekter Parameter der ADH-Wirkung an der Niere [43]. Bei Auftreten einer hypotonen Hyponatriämie ist die physiologische Reaktion eine Harnosmolalität < 100 mOsmol/kgH_2_O als Ausdruck für eine supprimierte ADH-Wirkung und daher maximale Wasserelimination. Ist die Harnosmolalität höher als 100 mOsmol/kgH_2_O, so besteht meistens eine ADH-Wirkung oder eine ADH-unabhängige Störung der Harndilution (z. B. unter einem Thiaziddiuretikum, höheres Alter, chronische Niereninsuffizienz). Nicht in allen Labors kann die Harnosmolalität (mittels eines Osmometers) gemessen werden. Eine gute Alternative ist die Berechnung der Harnosmolalität (Gl. 2). Diese korreliert sehr gut mit der gemessenen Harnosmolalität [46].

2 $$\begin{aligned}&\mathrm{H}\text{-Osmolalit{\"a}t}_{\mathrm{calc}}\left(\frac{\text{mOsmol}}{\text{kg}}\right)=\\&2*\left(\text{H-Na+K}\,\left(\frac{\text{mmol}}{\text{L}}\right)\right)+\\ &\frac{\mathrm{H}\text{-Harnstoff-}\mathrm{N}\,\left(\frac{\text{mg}}{\text{dL}}\right)}{2{,}8}+\frac{\mathrm{H}\text{-Glukose}\,\left(\frac{\text{mg}}{\text{dL}}\right)}{18}\end{aligned}$$

H‑Na+K…: Natrium und Kaliumkonzentration im Harn in mmol/l,
H‑Harnstoff-N…: Harnstoff-Stickstoffkonzentration im Harn in mg/dl,
H‑Glukose…: Glukosekonzentration im Harn in mg/dl (für weitere Details s. auch Supplement 2).

Eine Quantifizierung der renalen Wasserelimination gelingt nur durch die Berechnung der elektrolytfreien Wasserclearance (EFWC) (Gl. 3). Ein positiver Wert zeigt eine renale Wasserelimination und damit eine adäquate renale Antwort auf eine Hyponatriämie – das S‑Na^+^ sollte steigen; z. B. in der Phase der Autokorrektur der Hyponatriämie (s. Abschnitt „Überkorrektur“). Bei Polydipsie ist die EFWC typischerweise > 500 ml; aufgrund der hohen Trinkmenge steigt das S‑Na^+^ aber nicht an.

3 $$\begin{aligned}&\text{EFWC}\ (\mathrm{L})={}\\ &\text{Harnvolumen}\ (\mathrm{L})*\left(1-\frac{\text{H-Na+K}\ \left(\frac{\mathrm{mmol}}{\mathrm{L}}\right)}{\text{S-Na}\ \left(\frac{\mathrm{mmol}}{\mathrm{L}}\right)}\right)\end{aligned}$$

EFWC…: Elektrolyt freie Wasserclearance,
S‑Na…: Natriumkonzentration im Serum in mmol/L,
H‑Na+K…: Natrium- und Kaliumkonzentration im Harn in mmol/L.

#### d) Harn-Natriumkonzentration (mmol/l)

Die Harn-Natriumkonzentration (H-Na^+^) ist ein Parameter für das Ausmaß der renalen Natriumretention. Bei reduziertem EABV und daraus folgender hoher Aktivität des Renin-Angiotensin-Aldosteron-Systems (RAAS) und des Sympathikusnervensystems ist die H‑Na^+^ niedrig (< 30 mmol/l) [43]. Meist ist parallel dazu die Harnmenge gering (< 1 l/Tag). Die Wertigkeit der H‑Na^+^ ist in folgenden Konstellationen limitiert: Bei einer Polyurie/Polydipsie kann die H‑Na^+^ trotz Euvolämie auch unter 30 mmol/l liegen, v. a. wenn die Harnmengen sehr hoch sind. Eine H‑Na^+^ von > 30 mmol/l trotz einer Hypovolämie kann bei renalem Salzverlust (z. B. durch Diuretika, bei Nebennierenrindeninsuffizienz, unter RAAS-Blockade) oder bei einer metabolen Alkalose (bei metaboler Alkalose: Harn-Cl < 30 mmol/l) auftreten [3, 47].

#### e) Endokrinologisches Labor: ADH (Vasopressin)-Copeptin, Cortisol, TSH

Die Messung von ADH im Blut ist durch methodische Schwächen erheblich limitiert. Copeptin ist Teil des Prä-pro-ADHs und deutlich stabiler als ADH selbst. Es lässt sich einfacher messen und korreliert sehr gut mit der ADH-Konzentration. Dennoch ist die Wertigkeit der Bestimmung von Copeptin zur Differenzialdiagnose der Hyponatriämie gering und spielt deshalb in der klinischen Routine keine Rolle [48]. Niedrige Copeptinkonzentrationen trotz Hyponatriämie findet man bei Polydipsie und nephrogenem SIAD [10]. Im klinischen Alltag wird die ADH-Aktivität meist indirekt mittels der Harnosmolalität abgeschätzt. Möchte man nachträglich eine Einschätzung der Harnosmolalität vornehmen, erlaubt das spezifische Gewicht (Bestandteil konventioneller Teststreifen) einen groben Rückschluss, ist aber der Messung der Harnosmolalität deutlich unterlegen [49, 50]. Ein Wert < 1,005 entspricht einer Harnosmolalität von ca. < 150 mOsmol/kg (s. auch Supplement 2).

Eine wichtige Differenzialdiagnose in der Evaluierung von Patient:innen mit Hyponatriämie ist die primäre bzw. sekundäre Nebenniereninsuffizienz und – wenngleich sehr selten – die schwere Hypothyreose. Eine Messung von Cortisol basal (evtl. in Kombination mit Renin/Aldosteron) und TSH wird empfohlen, wenn eine laborchemische Konstellation eines SIAD vorliegt [10].

### C) Erhebung des Volumenstatus

Die Differenzialdiagnose und Therapie der Hyponatriämie orientiert sich am Volumenstatus der Patient:innen. Zur Evaluierung des Volumenstatus benötigen wir die Einschätzung sowohl des EABV als auch des Extrazellularvolumens (EZV). Für beide existieren keine exakten diagnostischen Parameter, weshalb nur eine Abschätzung des Volumenstatus möglich ist [51, 52]. Das EABV setzt sich zusammen aus: Herzzeitvolumen, peripherem Gefäßwiderstand und EZV. Für die Einschätzung des EABV bzw. des EZV werden klinische Parameter (z. B. Blutdruck, Puls, orthostatische Reaktion, trockene Axilla etc.) herangezogen und durch sonographische Parameter ergänzt [52–57]. Manchmal kann erst retrospektiv der Volumenstatus korrekt erhoben werden (z. B. durch Messung des S‑Kreatinins oder der Harnparameter nach Einleitung der Therapie) [58]. Grundsätzlich ist festzuhalten, dass die Wertigkeit der klinischen Untersuchung zur Beurteilung des Flüssigkeitshaushalts im Kontext der Hyponatriämie limitiert ist. Deshalb kann auch die Verfolgung entsprechender darauf basierender diagnostischer Pfade im Einzelfall in die Irre führen [51, 59].

#### a) Zeichen des verminderten EABV

Niedriger Blutdruck und hoher Puls (CAVE: bei arterieller Hypertonie in Relation zu den Basiswerten). Orthostatischer Abfall des Blutdruckes bzw. Anstieg des Pulses. Niedrige H‑Na^+^ (< 30 mmol/l) als Zeichen der maximalen RAAS-Aktivierung.

#### b) Zeichen des verminderten EZV

Klinik: trockene Schleimhäute, trockene Axilla. Sonographie: kollabierte V. cava inferior [60].

#### c) Zeichen des erhöhten EZV

Klinik: periphere Ödeme, Sonographie: dilatierte V. cava inferior ohne respiratorischer Variabilität des Durchmessers, hoher VExUS-Score, Pleuraerguss, multiple Kerley-B-Linien in der Lungensonographie [53].

## Ätiologien der Hyponatriämie

Zur Differenzialdiagnose der zugrunde liegenden Ätiologien der Hyponatriämien werden die S‑Osmolalität, H‑Osmolalität, H‑Natrium und Volumenstatus (EABV + EZV) herangezogen.

### Nichthypotone Hyponatriämien

Bei nichthypotonen Hyponatriämien ist die gemessene S‑Osmolalität > 275 mOsmol/kgH_2_O. In der klinischen Praxis werden diese am häufigsten bei hohen Blutzuckerspiegeln diagnostiziert.

#### Translokationale Hyponatriämien

Bei der translokationalen Hyponatriämie kommt es zur Akkumulation von osmotisch effektiven Substanzen wie Glukose, Mannit, Sorbit oder Kontrastmittel im Extrazellularraum. Dies führt zu einem osmotischen Shift von Wasser in den Extrazellularraum und somit zum Absinken des S‑Na^+^. Die S‑Osmolalität ist trotz Hyponatriämie normal oder erhöht. Es besteht somit kein „Laborfehler“ (Pseudohyponatriämie, s. unten), aber auch kein hypotoner Zustand, der eine entsprechende klinische Symptomatik erklären würde [10]. Am häufigsten führte eine schwere Hyperglykämie zur translokationalen Hyponatriämie. Der Effekt der Änderung des S‑Na^+^ durch den Anstieg der S‑Glukose kann abgeschätzt werden: Abfall des S‑Na+ um 2,4 mmol/l je 100 mg/dl Anstieg der S‑Glukose (Supplement 2) [61]. Die Genauigkeit dieser Berechnung sinkt mit steigendem Fettgehalt des Körpers und bei Blutzuckerkonzentration über 800 mg/dl [62].

#### Ineffektive Osmolyte

Harnstoff, Ethanol oder Methanol sind frei permeabel und werden deshalb als ineffektive Osmolyte bezeichnet. Akkumulieren diese Substanzen, so sind sie kausal *nicht* für eine Hyponatriämie verantwortlich. Es kann aber trotz Hyponatriämie die gemessene S‑Osmolalität normal oder erhöht sein. Die kalkulierte S‑Osmolalität (Supplement 2) korrigiert nur für den Harnstoff. Als Beispiel dafür kann bei Patienten mit sehr hohen BUN-Werten die gemessene Osmolalität trotz Hyponatriämie > 275 mOsmol/kgH_2_O sein. Ist die berechnete S‑Osmolalität < 275 mOsmol/kg so besteht dennoch eine hypotone Hyponatriämie. Die gemessene S‑Osmolalität kann ebenso für Ethanol (jedoch nicht für Methanol) korrigiert werden (Supplement 2) [63].

#### Pseudohyponatriämien

Eine Pseudohyponatriämie bezeichnet eine falsch niedrige Bestimmung des S‑Na^+^. Im Routinelabor erfolgt zumeist die indirekte Messung des S‑Na^+^ mit einer ionenselektiven Elektrode. Diese Methode benötigt einen Verdünnungsschritt vor der Messung des S‑Na^+^. Es kalkuliert dabei mit einer konstanten Menge an Plasmawasser (93 %, der Rest sind Proteine und Lipide). Durch eine hohe Konzentration von Triglyzeriden oder Proteinen (z. B. schwere Hypertriglyzeridämie, Paraproteinämie) im Blut kommt es zu einer Verminderung des Anteils an Plasmawasser zugunsten der Protein- und Lipidkonzentration, was die Berechnung des S‑Na^+^ falsch niedrig macht. Die gemessene S‑Osmolalität bleibt jedoch normal. Bei Messung mit einem Blutgasanalysegerät (direkte Messung mit ionenselektiver Elektrode) wird in diesem Fall der korrekte S‑Natriumwert angezeigt [64].

### Hypotone (echte) Hyponatriämien

#### Hyponatriämie (+Harn-Osmo < 100 mOsmol/kgH_2_O)

##### Polydipsie

Die zentrale Regulation des Durstempfindens und des Durstsättigungsgefühls ist ein sehr komplexer Prozess, aber ein wesentlicher Faktor für die Entstehung einer Hyponatriämie. Bei niedriger S‑Osmolalität sollte es eigentlich zu einem Rückgang des Durstgefühls kommen [65]. Bei der Polydipsie werden typischerweise sehr große Mengen getrunken (> 40–50 ml/kg/Tag), bedingt durch eine Veränderung des Durstempfindens bzw. des Durstsättigungsgefühls [66, 67]. Es entwickelt sich deshalb auch eine Polyurie (mit niedriger Harnosmolalität < 100 mOsmol/kgH_2_O). Primäre Polydipsien werden gehäuft bei psychiatrischen Erkrankungen (z. B. Schizophrenie) diagnostiziert. Davon abzugrenzen sind die *dipsogenen Polydipsien*, die sich nicht auf psychiatrische Erkrankungen zurückführen lassen. Einerseits können somatische Veränderungen im Durstzentrum (z. B. Tumor), andererseits habituelle Trinkgewohnheiten (viel Trinken als Lifestyle) dafür verantwortlich sein [67]. Sekundäre Polydipsien sind davon abzugrenzen. Diese entstehen z. B. bei ADH-Resistenz und führen in der Regel nicht zu einer Hyponatriämie. Eine Polydipsie ist häufig mit anderen Risikofaktoren für Hyponatriämien (z. B. „low solute intake“, s. unten) assoziiert. Bei typischer Anamnese einer psychogenen Polydipsie, jedoch erhöhter Harnosmolalität ist an die Kombination mit einem (evtl. durch Medikamente oder exzessiven Sport bedingtes) SIAD zu denken [68]*.*

##### „Low solute intake“

Die renale Kapazität, Wasser auszuscheiden, beträgt bei vollständiger Suppression von ADH und normaler Nierenfunktion bis zu 16 l/Tag oder mehr. Die maximale Menge an Wasser, die tatsächlich ausgeschieden werden kann, hängt aber von der Zufuhr an osmotisch aktiven Teilchen (v. a. Na^+^, K^+^) und Eiweiß (bzw. Aminosäuren), welche zu Harnstoff metabolisiert werden, ab. Die normale, im 24-h-Harn gemessene Ausscheidung an osmotischen Substanzen (= H-Osmolalität [mOsmol/kgH_2_O]*Harnmenge [l]), beträgt in etwa 10 mOsmol/kg/Tag. Somit kann bei einem 80 kg schweren Menschen (durchschnittliche tägliche Zufuhr ca. 800 mOsmol) und einer minimalen Harnosmolalität von 50 mOsmol/kgH_2_O (= minimale Verdünnungskapazität der menschlichen Niere, d. h. die Niere braucht zumindest 50 mOsmol osmotisch wirkender Substanzen, um 1 l Wasser ausscheiden zu können) ca. 16 l Wasser getrunken und auch wieder ausgeschieden werden, ohne dass es zu einer Hyponatriämie kommt [69]. Bei Menschen mit Essstörungen, älteren Personen oder chronischem Alkoholkonsum beträgt die tägliche Zufuhr an osmotischen Teilchen oft weniger als 300 mOsmol, sodass bei entsprechend hoher Trinkmenge eine Hyponatriämie entstehen kann [70, 71]. Eine „low solute intake“ ist aber auch ein entscheidender Kofaktor für die Entstehung vieler anderer Formen der Hyponatriämie [72].

##### Transientes SIAD

Als transientes SIAD werden jene Formen des SIAD bezeichnet, bei denen es rasch (nach Behandlung des Auslösers) oder zum Teil spontan zum Abfall des ADH-Spiegels kommt. Zum Zeitpunkt der Evaluierung der Hyponatriämie liegt die Harnosmolalität bereits unter 100 mOsmol/kgH_2_O, sodass das vorangehende SIAD nicht erkannt wird. Die Anamnese kann jedoch wegweisend in der Diagnose sein: Typische Situationen sind postoperativ (Stress und Schmerz), nach starker Übelkeit/Erbrechen oder herausfordernder sportlicher Betätigung (siehe exercise associated hyponatremia) [73].

#### Hyponatriämie (+Harn-Osmo > 100 mOsmol/kgH_2_O + Harn-Na^+^ > 30 mmol/l)

##### Syndrom der inadäquaten Antidiurese(SIAD)-Euvolämie

Das SIAD ist die häufigste Ursache für eine Hyponatriämie (ca. 35–40 %) [10]. Es bezeichnet alle Zustände, bei denen eine ADH-Sekretion nicht durch eine Hyperosmolalität oder Verminderung des EABV bedingt ist, bzw. alle Zustände einer inadäquat erhöhten ADH-Wirkung und ist somit eine Ausschlussdiagnose, das EZV ist normal. Wir verwenden den Begriff SIAD im Gegensatz zum Begriff des Syndroms der inadäquaten ADH-Sekretion (SIADH), da es auch Erkrankungen mit hoher ADH-Wirkung, aber niedrigen/normalen ADH-Spiegeln gibt [74, 75]. Die diagnostischen Kriterien für das SIAD sind in Tab. 2 dargestellt.

**Tab. 2:** Diagnostische Kriterien SIAD [10, 76]

1. Verminderte Serumosmolalität: S‑Osmolalität < 275 mOsmol/kgH_2_O
2. Inadäquat hohe Harnosmolalität: Harnosmolalität > 100 mOsmol/kgH_2_O
3. Klinische Einschätzung einer Euvolämie (= kein Hinweis auf Hypovolämie oder Hypervolämie) 4. Harn-Natriumkonzentration > 30 mmol/l bei normaler Salz- und Wasserzufuhr
5. *Ausschluss von: Glukokortikoidmangel und schwerer Hypothyreose 6. *Normale Nierenfunktion, keine rezente Einnahme von Diuretika (v. a. Thiazide)

Weitere biochemische Hinweise können sein: niedrige S‑BUN und S‑Harnsäurewerte sowie eine FE_Harnsäure_ > 12 % (Supplement 3) [77]

*Punkt 5 und 6: die Kriterien 1–4 können auch bei einem Glukokortikoidmangel, einer schweren Hypothyreose sowie bei rezenter Einnahme von Diuretika (v. a. Thiaziddiuretika) oder einer Niereninsuffizienz (eGFR < 45 ml/min) erfüllt sein [1, 10, 76]. Diese Situationen werden „traditionell“ als mögliche Ursachen für eine inadäquate Antidiurese vor der Diagnose eines SIAD ausgeschlossen

Es gibt eine Vielzahl möglicher Ursachen für ein SIAD (Tab. 3). Am häufigsten sind Malignome und Medikamente (jeweils ca. 20 %) gefolgt von pulmonalen bzw. ZNS-Erkrankungen (jeweils ca. 10 %). Je nach Studie findet sich aber bei bis zu 30 % der Patient:innen kein Auslöser für das SIAD [10]. Aufgrund dieser Pathomechanismen ist eine bildgebende Diagnostik zu Abklärung der Ätiologie zu empfehlen, falls die Ursache nicht auf der Hand liegt (CT-ZNS, Lunge und evtl. Abdomen). Auch wenn für die Diagnose eines SIADs formal das Fehlen einer Therapie mit Thiaziddiuretika gefordert ist, trifft man im Alltag auf Szenarien, in denen diese beiden Faktoren koinzidieren (Bsp.: Patient mit Hypertonie unter Thiazid, der davon unabhängig ein paraneoplastisches SIAD entwickelt). In solchen Fällen persistiert die Hyponatriämie nach Absetzen des Diuretikums, und eine weiterführende Abklärung ist angezeigt.

**Tab. 3:** Ursachen für ein SIAD [1, 3]

Zentralnervöse Erkrankungen	Infektion (Meningitis, Abszess, Enzephalitis) Inflammation (SLE, GBS, MS) Gefäßsystem (Insult, SAB, SDH) Trauma
Maligne Erkrankung	ZNS, Lunge, Oropharynx, Urogenitaltrakt, Lymphom, Gastrointestinaltrakt
Lungenerkrankungen	Infektionen: Pneumonie, Pneumonitis, TBC, COVID-19 Mechanisch: Beatmung, COPD, ARDS
Transiente ADH-Sekretion	Stress, Übelkeit, Erbrechen, Schmerz, postoperativ Starke sportliche Belastung
Hereditär	Nephrogenes SIAD
Medikamente	Siehe Tab. 4
Unbekannte Ätiologie	–

*SIAD* Syndrom der inadäquaten Antidiurese, *SLE* systemischer Lupus erythematodes, *GBS* Guillain-Barré-Syndrom, *MS* multiple Sklerose, *SAB* subarachnoidale Blutung, *SDH* subdurales Hämatom, *ZNS* zentrales Nervensystem, *TBC* Tuberkulose, *COPD* chronisch obstruktive Lungenerkrankung, *ARDS* „adult respiratory distress syndrome“

#### Spezielle Formen des SIAD

##### Medikamentös bedingte Hyponatriämien

Medikamente sind eine der häufigsten Ursachen von Hyponatriämien. Diese können die zentrale ADH-Sekretion stimulieren, die Wirkung von ADH an der Niere potenzieren, direkt den Vasopressinrezeptor an der Niere stimulieren oder die osmotische Schwelle zur ADH-Sekretion in Richtung niedrigere S‑Osmolalität verschieben (Tab. 4). Die häufige Form einer Diuretika-bedingten Hyponatriämie wird weiter unten gesondert besprochen. Im Umkehrschluss darf jedoch nicht verfrüht auf eine medikamentöse Ursache geschlossen werden, ohne dass eine sorgfältige Abklärung hinsichtlich anderer Ätiologien erfolgt ist.

**Tab. 4:** Auswahl an Medikamenten, welche ein SIAD verursachen können [10, 78, 79]

Verstärkung der Wirkung von ADH an der Niere oder direkte Stimulation des V2R	Antidepressiva Antiepileptika Antipsychotika Chemotherapeutika Diverses	SSRI, NRI, trizyklische Antidepressiva, MAO-Hemmer Carbamazepin, Valproinsäure, Lamotrigin Haloperidol, Chlorpromazin Cyclophosphamid, Melphalan, Methotrexat Tramadol, Ecstasy, Amiodaron, NSAR, ACE-Hemmer (selten), PPI, Clofibrate etc.
Stimulation der ADH-Sekretion	Chemotherapeutika	Vincristin, Ifosfamid
ADH (Vasopressin)-Analoga	ADH (Vasopressin)-Analoga	Desmopressin, Vasopressin, Terlipressin (selten, da selektive Wirkung am V1R), Oxytocin
Reset-Osmostat	–	Carbamazepin, Venlafaxin

*V1R* bzw. *V2R* Vasopressin-1 bzw. -2 (ADH)-Rezeptor; *SSRI* selektiver Serotoninwiederaufnahmehemmer, *NRI* Noradrenalinwiederaufnahmehemmer, *MAO* Monoaminoxidase, *NSAR* nichtsteroidale Antirheumatika, *ACE* „angiotensin-converting enzyme“, *PPI* Protonenpumpenhemmer

##### Reset-Osmostat/Barostat

Der Reset-Osmostat bezeichnet einen Zustand mit der Verschiebung der osmotischen Schwelle der ADH-Sekretion hin zu niedrigerem S‑Na^+^ (bzw. S‑Osmolalität) und ist eine Variante des SIAD [80]. Fällt die S‑Na^+^-Konzentration unter diese Schwelle ab, kommt es jedoch zur vollständigen Unterdrückung der ADH-Sekretion, sodass das S‑Na^+^ trotz weiterer Wasserbelastung nicht weiter absinkt. Die niedrigsten S‑Na^+^-Konzentrationen liegen meist um die 125–130 mmol/l, die Harnosmolalität ist erhöht, sinkt aber nach weiterer Wasserbelastung (s. Wasserbelastungstest Supplement 6 [81]) auf < 100 mOsmol/kgH_2_O ab [82]. Die Prävalenz des Reset-Osmostat ist unklar. Häufig ist ein Reset-Osmostat passager nach schweren Infektionen oder ZNS/Lungenerkrankungen zu beobachten, wobei dieser Zustand auch über viele Monate persistieren kann. In der Schwangerschaft kommt es zu einem physiologischen Reset-Osmostat. Eine Therapie ist im Regelfall nicht notwendig, obwohl dies zum Teil kontroversiell diskutiert wird [83].

Der Reset-Barostat ist eine neu beschriebene Form des SIAD. Dabei kommt es zu einem linearen Abfall des ADH (Copeptins) in Relation zum Anstieg der S‑Osmolalität unter der i.v.-Gabe von NaCl 3 %. Es wird vermutet, dass die Schwelle der Barorezeptor-mediierten ADH-Sekretion in Richtung Euvolämie verschoben ist [80].

##### Hyponatriämie in der Schwangerschaft

In der Schwangerschaft kommt es zu relevanten Änderungen in der Regulation des Wasserhaushaltes. Die vermehrte Produktion von vasodilatatorischen Hormonen führt zu einer Barorezeptor-vermittelten vermehrten ADH-Sekretion, welche gemeinsam mit einer Absenkung der osmotischen Durstschwelle (*Reset-Osmostat*) zu einem physiologischen Abfall des S‑Na^+^ um 3–5 mmol/l führt [84]. Während der Schwangerschaft kann es zusätzlich zur nichtosmotischen Stimulation der ADH-Sekretion kommen (Hyperemesis gravidarum: Übelkeit, Geburt: Schmerz), welche bei inadäquat hoher Zufuhr von hypotonen Lösungen zu schweren Hyponatriämien führen kann [85, 86]. Des Weiteren findet sich in bis zu 15 % der Patientinnen mit Präeklampsie eine Hyponatriämie, die als zusätzliches Kriterium für den Schweregrad der Erkrankung herangezogen wird [87].

##### Nephrogenes SIAD

Dabei handelt es sich um eine extrem seltene, X‑chromosomal vererbte Gain-of-Function-Mutation des Arginin-Vasopressin-Rezeptor 2(*AVPR2*)-Gens, welche trotz adäquat niedriger ADH-Spiegel zu einer permanenten Stimulation des AVPR2 führt. Der Phänotyp ist sehr heterogen und reicht von schweren Hyponatriämien in der Kindheit bis zu milderen Formen, welche nur nach Wasserbelastungstests (Supplement 6) erkannt werden [74, 88].

##### Hyponatriämie nach Hypophysenoperationen

Durch die Manipulation an der Hypophyse kann es nach initialem ADH-Mangel mit Polyurie zu einer passageren (meist zwischen Tag 6 und 14 postoperativ) vermehrten (unkontrollierten) ADH-Sekretion kommen. Diese kann später, wenn der Hypophysenstiel durchtrennt wurde und alle ADH-Reserven im Hypophysenhinterlappen aufgebraucht sind, in einen ADH-Mangel übergehen („triphasic response“). Bei erhaltenem Hypophysenstiel hingegen klingt das SIAD nach ein paar Tagen spontan ab („biphasic response“) [89].

##### Hyponatriämie bei Hypothyreose

Eine schwere Hypothyreose führt zum Abfall der GFR, reduziert das Herzminutenvolumen und den Blutdruck und vermindert damit die Gesamtkapazität der Niere, Wasser auszuscheiden. Dies begünstigt das Auftreten einer Hyponatriämie, ist aber selbst beim Myxödem nur selten der alleinige Auslöser einer Hyponatriämie. Bestehen eine Hypothyreose und Hyponatriämie, so muss immer nach zusätzlichen Ursachen für die Hyponatriämie gesucht werden, v. a. nach einer Nebennierenrindeninsuffizienz [90, 91].

##### „Excercise associated hyponatremia“ (EAH)

Die Exercise-associated-Hyponatriämie (EAH) wird definiert als das Auftreten einer Hyponatriämie innerhalb eines Zeitraumes von 24 h nach körperlicher Anstrengung, es handelt sich somit um eine akute Hyponatriämie [92]. Sie wird beobachtet im Rahmen außerordentlicher körperlicher Anstrengung mit Verlust großer Mengen Schweiß, welcher gleichzeitig mittels relativ hypotoner Flüssigkeiten ersetzt wird. Parallel dazu besteht eine starke ADH-Ausschüttung durch den extremen Ausdauersport [93]. Insbesondere beim Marathonlauf, aber auch bei anderen Extremsportarten wurde diese Form der Hyponatriämie mit teils schwerer Ausprägung beschrieben [13, 94, 95]. Zu bemerken ist jedoch, dass nur ein geringer Anteil der bei einem Marathon kollabierten Sportler eine EAH aufweisen dürften [96]. Differenzialdiagnostisch ist immer an den Hitzschlag zu denken [93].

Symptome einer EAH reichen von einem Gefühl des Blähbauches, über Gewichtszunahme im Rahmen des Sports, fehlendem Durstgefühl bis hin zu Dyspnoe, weißlichem Sputum und neurologischen Beeinträchtigungen [93].

##### Renaler Salzverlust – Hypovolämie

- Diuretika-induzierte Hyponatriämie

Alle Diuretika induzieren eine Natriurese und führen damit potenziell zu einer Hypovolämie, aber nicht immer zu einer Hyponatriämie. Während Schleifendiuretika die Harnkonzentration (weniger aber die Harndilution) stören und deshalb häufiger zu einer Hypernatriämie führen, hemmen Thiaziddiuretika v. a. den Harndilutionsmechanismus und sind deshalb hauptverantwortlich für Diuretika-induzierte Hyponatriämien [97, 98]. Schleifendiuretika können über eine Hypovolämie ebenfalls zur Barorezeptor-mediierten Stimulation von Durstgefühl und ADH-Sekretion führen und damit eine Hyponatriämie verursachen. Auch die Kombination von Schleifendiuretika mit Thiaziddiuretika oder Aldosteronantagonisten erhöht das Risiko einer Hyponatriämie [99, 100].

Thiazid-induzierte Hyponatriämien können sowohl in einer hypovolämischen als auch SIAD-ähnlichen Form auftreten [76, 101]. Die Differenzialdiagnose ist schwierig und kann durch die Bestimmung der fraktionellen Exkretion von Harnsäure (< 12 % bei Hypovolämie) oder evtl. durch Berechnung anderer Parameter („strong ion difference“, Harn-Chlorid-Kalium) ermöglicht werden [77, 101]. Die euvolämische Form der Thiazid-assoziierten Hyponatriämie ist u. a. gekennzeichnet durch eine Störung der Harndilution, verstärktes Durstgefühl, vermehrte ADH-Wirkung am Sammelrohr sowie eine genetische Komponente und ein erhöhtes Risiko für eine Rezidivhyponatriämie nach Reexposition, weshalb diese Patienten niemals wieder ein Thiaziddiuretikum erhalten sollten [102]. Vor allem ältere Frauen weisen ein relativ hohes Risiko für diese Elektrolytstörung auf, weshalb nach Therapiebeginn eine kurzfristige Elektrolytkontrolle ratsam scheint [103]. Thiazid-induzierte Hyponatriämien treten meist im ersten Monat nach Therapiebeginn auf und können ausgeprägt sein [104].

- Hyponatriämie bei Niereninsuffizienz

Sinkt die GFR, nimmt naturgemäß auch die renale Kapazität zur Wasserelimination ab. Zusätzlich kann es bei Nierenerkrankungen zur Störung der Harndilutionsmechanismen kommen. Deshalb ist auch die Prävalenz an Dysnatriämien bei chronisch Nierenkranken höher [105]. Bei terminaler Niereninsuffizienz kann eine Hyponatriämie sowohl bei Hämodialyse und etwas häufiger bei Peritonealdialyse (bis 15 %) beobachtet werden und ist jeweils mit einer deutlich erhöhten Mortalität assoziiert [106, 107]. Die Ursachen sind meist eine in Relation zur Salzzufuhr höhere Zufuhr an freiem Wasser, welche mit einer Veränderung des Durstempfindens zum Teil erklärt werden kann, sowie eine Abnahme der Restnierenfunktion [108, 109]. Bei Peritonealdialyse kann auch die (exzessive) Verwendung von Icodextrin zu einer Hyponatriämie führen. Dabei führt die Akkumulation von Maltose im Serum zu einer translokationalen Hyponatriämie [110].

- Hyponatriämie bei Nebennierenfunktionsstörung

Die primäre Nebenniereninsuffizienz kennzeichnet einen Mangel an Cortisol und Aldosteron. Der Aldosteronmangel führt über einen renalen Salzverlust zur Hypovolämie und damit zu einer Barorezeptor-vermittelten Stimulation der ADH-Sekretion. Die primäre Nebenniereninsuffizienz präsentiert sich daher klinisch als renales Salzverlustsyndrom [111]. Zusätzlich führt der Cortisolmangel einerseits zu einer fehlenden physiologischen Suppression der ADH-Sekretion („Entzügelung“) und andererseits durch Effekte auf das Gefäßsystem zu einem Rückgang des Herzminutenvolumens bzw. Blutdruckabfall und damit ebenfalls zu einer Barorezeptor-mediierten ADH-Sekretion. Direkte Effekte auf das renale Gefäßsystem senken die GFR und stören damit die renale Wasserausscheidung zusätzlich. Ein isolierter Cortisolmangel (Hypophysenpathologie mit ACTH-Mangel und daraus resultierender sekundärer Nebenniereninsuffizienz) kann über diese Mechanismen auch bei normalem Aldosteron zu einer Hyponatriämie führen, welche aber biochemisch/klinisch mehr einem SIAD ähnelt und deshalb immer ausgeschlossen werden sollte [112]. In einer skandinavischen Studie (*n* = 247) bestand bei 84 % der Patient:innen zum Zeitpunkt der Erstdiagnose einer Nebenniereninsuffizienz eine Hyponatriämie [113]. Die laborchemische Diagnostik des Hypocortisolismus ist schwierig, da die Cut-off-Spiegel vom verwendeten Assay abhängen und deshalb an das lokale Labor angepasst werden müssen. Im Allgemeinen definiert ein morgendlicher totaler Cortisolspiegel < 5 µg/dl bzw. von < 18 µg/dl nach einer Stimulation mit ACTH (Synacthen-Test) einen Hypocortisolismus [114]. In einer Studie mit hyponatriämischen Patient:innen konnte hingegen nur ein morgendlicher Cortisolspiegel > 10,9 µg/dl einen Hypocortisolismus mit hoher Wahrscheinlichkeit ausschließen [112]. Onkologische Patient:innen mit Hyponatriämie, welche unter einer Therapie mit Checkpointinhibitoren stehen, sollten gezielt auf einen Hypocortisolismus untersucht werden, weil unter diesen Medikamenten relativ häufig eine Hypophysitis auftritt, allerdings auch eine Adrenalitis, sodass zwischen primärer und sekundärer Nebenniereninsuffizienz unterschieden werden muss [115].

- Andere Salzverlustsyndrome

Neben dem Hypoaldosteronismus können die sehr seltenen monogenetischen Erkrankungen des Mineralkortikoidrezeptors oder des epithelialen Natriumkanals im distalen Nephron ebenfalls zu Hyponatriämien führen (Pseudohypoaldosteronismus Typ 1a und 1b) [116]. Diese renalen Salzverlustsyndrome führen über die schwere Hypovolämie zur ADH-Sekretion und Hyponatriämie. Zusätzlich zeigen die Betroffenen eine Hyperkaliämie und metabole Azidose. Andere hereditäre renale Salzverlustsyndrome führen hingegen nicht zu Hyponatriämien, mit Ausnahme des Gitelman-Syndroms [117, 118]. Ein hyporeninämischer Hypoaldosteronismus führt ebenfalls nicht zu einer Hyponatriämie [76, 119].

Über die Existenz bzw. Relevanz des zerebralen Salzverlustsyndroms (CSW) herrscht in der Literatur große Uneinigkeit. Die pathophysiologische Basis für das CSW sind die zerebrale Produktion eines natriuretischen Faktors sowie eine verminderte Stimulation der Niere durch das sympathische Nervensystem und eine fehlende Aldosteronproduktion trotz Hypovolämie. Biochemisch kann bei bestehender Hyponatriämie ein CSW nicht vom SIAD unterschieden werden [120–122]. In gewissen Situationen, wie nach neurochirurgischen Eingriffen, kann manchmal ein passageres zerebrales Salzverlustsyndroms nicht ausgeschlossen werden [123].

#### Hyponatriämie (+Harn-Osmo > 100 mOsmol/kgH_2_O + Harn-Na^+^ < 30 mmol/l)

Bei Herz‑, Leber und Niereninsuffizienz sind hypervolämische Hyponatriämien am häufigsten anzutreffen. Wichtig ist, dass bei all diesen Entitäten prinzipiell auch eine hypovolämische Hyponatriämie auftreten kann, welche dieselbe biochemische Konstellation zeigt und nur anhand der Anamnese und Klinik unterschieden werden kann [124].

##### Hyponatriämie bei Herzinsuffizienz – Hypervolämie

Bei dekompensierter Herzinsuffizienz ist die Prävalenz der Hyponatriämie mit bis zu 20 % sehr hoch. Der Hauptmechanismus ist die Barorezeptor- und Angiotensin-II-vermittelte Stimulation der ADH-Sekretion und des Durstempfindens. Zusätzlich stört der Abfall der GFR und die vermehrte Na-Resorption am proximalen Tubulus die maximale Wasserausscheidungskapazität der Nieren [124].

Aber auch unter einer dekongestiven Therapie können Hyponatriämien entstehen. Die Behandlung mit Diuretika, welche am distalen Tubulus wirken (Thiazide, Aldosteronantagonisten) und somit die Harndilution stören, können zum Abfall des S‑Na^+^ führen [125]. Die Veränderung des S‑Na^+^ ist bei sequenzieller Nephronblockade schwierig abzuschätzen. Die Kombination von Furosemid mit Acetazolamid dürfte im Gegensatz zur Kombination mit Thiaziddiuretika zu einem geringeren Risiko für die Entwicklung einer Hyponatriämie führen [99, 126].

##### Hyponatriämie bei Lebererkrankungen – Hypervolämie

Die Prävalenz der Hyponatriämie ist, v. a. bei dekompensierter Leberzirrhose, mit 20–60 %% sehr hoch. Eine niedrige Natrium-Konzentrationen ist ein prädiktiver Faktor für den Schweregrad der Erkrankung, dem Auftreten von Komplikationen wie einer hepatischen Enzephalopathie oder Aszites und der Mortalität (Na-MELD-Score) [127–129]. Die Pathogenese der Hyponatriämie beginnt mit der Entwicklung einer portalen Hypertension, welche sekundär zur venösen Dilatation im Splanchnikussystem führt. In der Folge muss das Herzminutenvolumen erhöht werden, um den Blutdruck aufrechtzuerhalten. Versagt diese Kompensation, so sinkt der Blutdruck (Verminderung des EABV), und es kommt wie bei der Herzinsuffizienz zur Stimulation des sympathischen Nervensystems und des RAAS über Barorezeptoren sowie der nicht-osmotischen Stimulation der ADH-Sekretion und somit zur vermehrten renalen Wasserretention [128]. Zusätzlich haben bis zu 50 % der Patient:innen mit Leberzirrhose eine Nebennierenrindeninsuffizienz [128]. Die Behandlung mit Terlipressin, einem synthetischen ADH-Analogon, welches zur Therapie des hepatorenalen Syndroms eingesetzt wird, kann theoretisch das Risiko für eine Hyponatriämie erhöhen. In der rezenten CONFIRM-Studie (Studie zur Wirksamkeit und Sicherheit von Terlipressin und Albumin für die Behandlung des hepatorenalen Syndroms) konnte jedoch kein erhöhtes Risiko beobachtet werden [130, 131]. Auch Pseudohyponatriämien bei cholestatischen Lebererkrankungen (durch Cholesterin bzw. Lipoprotein X) sind beschrieben [132].

##### Hyponatriämie bei nephrotischem Syndrom – Hypervolämie

Hyponatriämien bei nephrotischem Syndrom sind selten und v. a. bei Kindern beschrieben. Sie sind durch eine Reduktion des EABV bei schwerer und rasch einsetzender Hypoalbuminämie in Kombination mit einem Abfall der GFR bedingt [76].

##### Hypovolämische Formen der Hyponatriämie

Eine hypovolämische Hyponatriämie entsteht durch einen Verlust von Natrium und einer damit verbundenen Reduktion des EABV. Dies führt zu einer Barorezeptor-mediierten Stimulation der ADH-Sekretion. Durch einen erhaltenen Durstmechanismus kommt es zu einem in Relation höheren Verlust von Natrium als Wasser, was letztendlich die Hyponatriämie bedingt. Die niedrige H‑Na^+^ von < 30 mmol/l (aufgrund der RAAS-Stimulation durch ein vermindertes EABV) in Kombination mit einer Harnosmolalität > 100 mOsmol/kgH_2_O bei klinischer Hypovolämie zeigt typischerweise einen extrarenalen Volumenverlust (v. a. Darm) an, kann aber auch nach Absetzen von zuvor eingenommenen Diuretika, im Sinne einer Diuretika-induzierten Hyponatriämie, beobachtet werden. Unter laufender Diuretikatherapie kann die H‑Na^+^ trotz Hypovolämie hingegen auch über 30 mmol/l liegen.

#### Multifaktorielle Hyponatriämie

Eine Hyponatriämie entsteht oft durch mehrere Faktoren [133]. Es sollen hier einige typische Situationen angeführt werden, bei denen multifaktorielle Ursachen für eine Hyponatriämie charakteristisch sind:

Extrarenale Flüssigkeitsverluste (hypotone Körperflüssigkeiten wie bei Diarrhö oder Fieber) können nur bei hoher oraler Flüssigkeitszufuhr und niedriger Zufuhr an osmotisch aktiven Substanzen (Salz und Proteine) in Kombination mit einer Barorezeptor-mediierten ADH-Sekretion zu einer Hyponatriämie führen [76]. Eine ähnliche Situation kann auch bei der Vorbereitung zur Koloskopie entstehen. Hier besteht die Kombination aus Polydipsie, „low solute intake“ und einer nicht-osmotischen Stimulation von ADH (z. B. durch Stress, Schmerz, Übelkeit) [134]. Bei psychiatrischen Patient:innen mit Polydipsie ist die Harnosmolalität nicht immer < 100 mOsmol/kgH_2_O, da viele Psychopharmaka die ADH-Sekretion/-wirkung erhöhen können [67]. Manchmal kann auch eine nicht erkannte Nebenniereninsuffizienz bei anderen Risikofaktoren für eine Hyponatriämie (Leberzirrhose, Koloskopievorbereitung) relevant werden [135, 136]. Vor allem bei therapierefraktären Hyponatriämien sollte ein Hypocortisolismus immer ausgeschlossen werden [112]. Bei einer Polydipsie ist die Kombination mit „low solute intake“ häufig anzutreffen [137]. Prinzipiell sollten daher bei jeder Hyponatriämie die Trinkmenge und das Essverhalten sorgfältig evaluiert werden [72, 133].

## Algorithmus zur Differenzialdiagnose

**Abb. 2:**
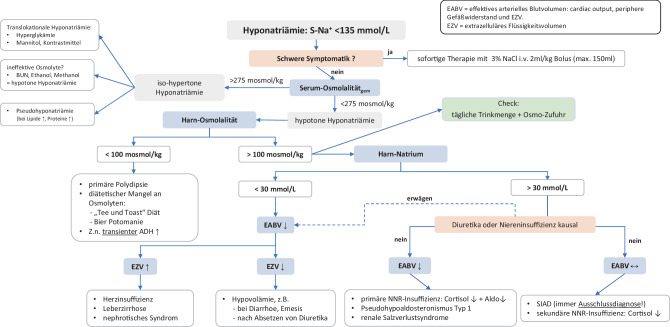
Algorithmus zur Differenzialdiagnose der Hyponatriämie

Nachdem die Hyponatriämie durch Messung des S‑Na^+^ diagnostiziert wurde, führt Abb. 2 durch den weiteren diagnostischen Algorithmus.

### Frage 1: Besteht eine schwere klinische Symptomatik?

**Abb. 3:**
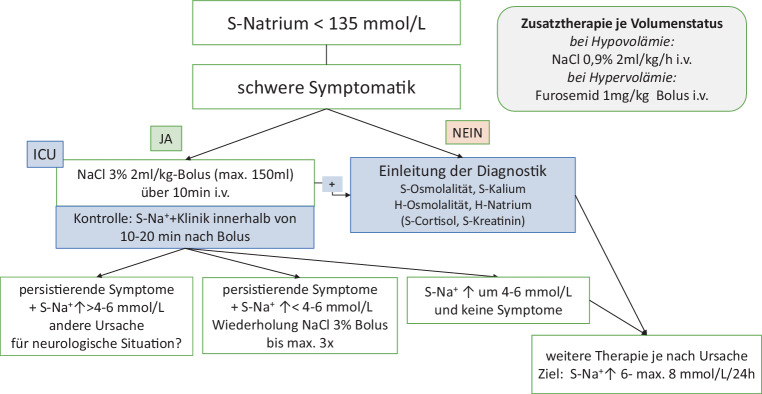
Therapie der symptomatischen chronischen/akuten Hyponatriämie

Besteht eine schwere klinische Symptomatik (s. klinischer Schweregrad), so muss sofort eine i.v.-Therapie mit NaCl 3 % eingeleitet werden (s. Abb. 3), ohne dass andere Laborparameter (z. B. S‑Osmolalität) abgewartet werden. NaCl 3 % kann über die periphere Vene infundiert werden [138].

### Frage 2: Wie hoch ist die gemessene Serumosmolalität?

A. > 275 mOsmol/kgH_2_O: es besteht eine isotone oder hypertone Hyponatriämie. Ursachen dafür können sein:
  - *Pseudohyponatriämie:* Das S‑Na^+^ wird im Labor bei Verwendung der indirekten Messmethode falsch gemessen. Die berechnete S‑Osmolalität ist < 275 mOsmol/kgH_2_O. In der Blutgasanalyse ist die Natriumkonzentration > 135 mmol/l.
  - *Translokationale Hyponatriämie:* Akkumulation von osmotisch wirksamen Substanzen wie Glukose, Mannit oder Röntgenkontrastmittel. Für Glukose wird bei der Berechnung der S‑Osmolalität korrigiert, weshalb auch die berechnete S‑Osmolalität > 275 mOsmol/kgH_2_O beträgt [139]. Akkumulieren andere osmotisch wirksame Teilchen (z. B. Mannit), so ist die berechnete S‑Osmolalität aber < 275 mOsmol/kgH_2_O.
  - *Akkumulation von ineffektiven Osmolyten* wie Harnstoff (Nierenversagen) oder Ethanol/Methanol. Wird die S‑Osmolalität berechnet, muss für diese Osmolyte korrigiert werden. Falls die korrigierte, berechnete S‑Osmolalität < 275 mOsmol/kgH_2_O ist, besteht eine *hypotone* Hyponatriämie (für Details s. Supplement 2).
  - Eine bestehende klinische Symptomatik ist durch eine isohypertone Hyponatriämie nicht zu erklären.
  B. < 275 mOsmol/kgH_2_O (= verminderte S‑Osmolalität): Dies ist die häufigste Kategorie. Es besteht eine echte (hypotone) Hyponatriämie, eine weitere Abklärung ist notwendig – weiter mit Frage 3.

### Frage 3: Wie ist die Harnosmolalität?

A. *<100* *mOsmol/kgH*_*2*_*O:* In diesem Fall bestehen eine physiologische, vollkommene Suppression der ADH-Sekretion und eine adäquate renale Reaktion auf die Hyponatriämie. Wegen einer altersbedingten Abnahme der renalen Fähigkeit zur Harndilution kann bei älteren Menschen trotz fehlender ADH-Sekretion die Harnosmolalität bei bis zu 150 mOsmol/kgH_2_O liegen.
  Mögliche Ursachen:
  - *Primäre Polydipsie:* Es besteht entweder eine psychiatrische Grunderkrankung oder eine dipsogene Polydipsie durch habituelles Trinken oder eine somatische Störung des Durstzentrums.
  - „*Low solute intake*“: Eine niedrige Zufuhr an osmotisch wirksamen Substanzen über die Nahrung (Na^+^, K^+^, Protein) limitiert die renale Kapazität, Wasser auszuscheiden, und kann ein isolierter, häufiger ein Kofaktor für das Auftreten einer Hyponatriämie sein [69, 72]. Die ausgeschiedene Menge an osmotisch aktiven Substanzen im Harn ist in diesen Fällen meist < 300 mOsmol/Tag.
  - *Transiente ADH-Ausschüttung*: Zum Zeitpunkt der Diagnostik ist der ADH-Stimulus nicht mehr vorhanden, er lässt sich aber aus der Anamnese erheben. Typische transiente ADH-Stimulationen sind durch Schmerzen, Übelkeit oder Erbrechen (z. B. post-OP) bedingt [10].
B. *>* *100* *mOsmol/kgH*_*2*_*O:* Trotz einer verminderten S-Osmolalität besteht eine inadäquate Harnkonzentrierung, welche, bis auf wenige Ausnahmen, durch eine erhöhte ADH-Sekretion bedingt ist – deshalb muss zur weiteren Differenzialdiagnose als Nächstes die H‑Na^+^ gemessen werden.

### Frage 4: Wie hoch ist die Harn-Natriumkonzentration und wie ist das EZV?

A. *Harn-Na*^*+*^* <* *30* *mmol/l:* Bei einer Verminderung des EABV kommt es zu einer Stimulation des RAAS, des sympathischen Nervensystems und von ADH. Diese bewirken eine maximale Stimulation der renalen Natriumretention, sodass die H‑Na^+^ auf < 30 mmol/l abfällt und simultan die Harnmenge reduziert ist (ca. < 1000 ml/Tag).
  - *+* *EZV erhöht:* Bei Leber‑, Herzinsuffizienz oder nephrotischem Syndrom besteht der Zustand eines niedrigen EABV, aber erhöhten EZV, was sich klinisch durch Aszites, Ödembildung oder eine dilatierte V. cava inf. zeigt.
  - *+* *EZV vermindert:* Bei Verlust von iso-hypotonen Körperflüssigkeiten (Diarrhö) kommt es zu einem Volumenmangel, falls dieser > 10 % des EZV beträgt, ist die Folge eine starke ADH-Sekretion. Die Volumen-bedingte ADH-Sekretion führt gemeinsam mit der Zufuhr an hypotonen Lösungen (Trinken, Glukose 5 %) zur Hyponatriämie.
B. *Harn-Na*^*+*^* >* *30* *mmol/l:* Entweder es fehlt eine RAAS-Stimulation, weil das EABV normal/erhöht ist, oder das RAAS kann seinen Effekt auf die renale Natriumretention nicht umsetzen: Bei einem Harn-Na^+^ > 30 mmol/l muss deshalb zuerst eine rezente Diuretikaeinnahme bzw. eine Niereninsuffizienz ausgeschlossen werden. In diesen Fällen korreliert das H‑Na^+^ nicht mit dem EABV. Es könnte auch eine Erkrankung vorliegen, bei der ein vermindertes EABV normalerweise mit einem H‑Na^+^ < 30 mmol/l einhergeht (s. Frage 4, Punkt A).
  - *+* *EABV vermindert*: Es besteht eine Störung der renalen Natriumretention. Ursachen können ein renal-tubulärer Defekt per se sein oder eine Störung der hormonellen Regulation bzw. Wirkung auf die renalen Natriumretention: Hypoaldosteronismus, renale Salzverlustsyndrome.
  - *+* *EABV normal/erhöht:* Diese Konstellation entspricht dem Syndrom der inadäquaten ADH-Wirkung (SIAD) oder einer sekundären Nebennierenrindeninsuffizienz.

### Frage 5: Wie hoch ist die tägliche Zufuhr an Flüssigkeiten und Osmolyten?

Für alle Hyponatriämien ist die tägliche Flüssigkeitszufuhr von entscheidender Bedeutung; sowohl für die Genese als auch für das Ausmaß der Hyponatriämie. Durch Trinken wird eine nahezu elektrolytfreie Lösung zugeführt. Kann diese nicht renal eliminiert werden, sinkt das S‑Na^+^. Je höher die Trinkmenge, umso eher entsteht eine Hyponatriämie [140]. Dasselbe gilt für die i.v.-Zufuhr von Glukose 5 %. Da Glukose im Körper umgehend metabolisiert wird, entspricht Glukose 5 % der Zufuhr einer elektrolytfreien Lösung. Selbst wenn isotones NaCl (Osmolalität 308 mOsmol/kgH_2_O) infundiert wird, kann das S‑Na^+^ weiter abfallen, wenn die Harnosmolalität (oder exakter Na^+^+K^+^-Konzentration im Harn) > 300 mOsmol/kgH_2_O (oder Na^+^+K^+^ > 154 mmol/l) ist (z. B. SIAD).

Eine ausreichende Zufuhr an Osmolyten (Na^+^, K^+^, Protein) ist für die renale Wasserausscheidungskapazität essenziell. Wurde in den letzten Tagen vor der Diagnose einer Hyponatriämie wenig gegessen, so kann dies ein weiterer Faktor für die Genese der Hyponatriämie sein (s. „low solute intake“).

### Limitationen des Algorithmus

Bei Verwendung des Algorithmus muss man sich bewusst sein, dass dieser gewissen Limitation unterliegt:

Wann immer möglich, sollten die Harnindizes (Osmolalität und Elektrolyte aus dem Spontanharn) *vor *Einleitung einer Therapie bestimmt werden (Ausnahme: symptomatische Patient:innen), da ansonsten die diagnostische Wertigkeit erheblich eingeschränkt sein kann. Die Harnindizes repräsentieren immer den aktuellen Zustand der renalen Regulation und erklären nicht immer den Grund für das Auftreten einer Hyponatriämie, z. B. findet schon eine Gegenregulation statt?

Eine korrekte klinische Einschätzung des EABV ist nicht immer möglich.

Bestehen mehrere kausale Faktoren für das Auftreten einer Hyponatriämie, welche zudem die biochemischen Parameter der Differenzialdiagnose beeinflussen (z. B. Niereninsuffizienz, Diuretikatherapie, Herzinsuffizienz und Polydipsie) kann der Algorithmus die Kausalität der Hyponatriämie nicht eindeutig erkennen. Manchmal kann erst retrospektiv, nach Einleitung einer Therapiemaßnahme zur Behandlung der wahrscheinlichsten Ursache, (z. B. kardiale Dekompensation – Intensivierung der Diuretika) der eigentliche Grund für die Hyponatriämie erkannt werden.

## Therapie

Das primäre Ziel der Therapie der Hyponatriämie ist eine Normalisierung des S‑Na^+^, um vorhandene Symptome, bedingt durch die Hyponatriämie, zu therapieren bzw. um das Auftreten dieser Symptome bei milder Hyponatriämie zu verhindern. Eine Normalisierung des S‑Na^+^ reduziert die Mortalität und verkürzt den Krankenhausaufenthalt [141, 142]. Selbst bei milder neurokognitiver Symptomatik kann diese verbessert werden, wenn das S‑Na^+^ erhöht wird [4]. Es gibt jedoch nur wenige prospektive Studien hinsichtlich der Effektivität ausgewählter Therapien in Bezug auf Mortalität und Morbidität [143–145]. Die Ergebnisse einer relevanten Therapiestudie sind noch ausständig [146]. Gleichzeitig steht die Vermeidung therapieassoziierter Nebenwirkungen und Komplikationen im Vordergrund.

### Die Bedeutung der zeitlichen Entwicklung einer Hyponatriämie

Eine Hyponatriämie gilt per definitionem dann als akut, wenn sie nachweislich innerhalb eines Zeitraumes von 48 h aufgetreten ist (basierend auf Labormessungen) oder dies aus der Vorgeschichte mit hoher Wahrscheinlichkeit angenommen werden kann (z. B. EAH) [2, 31, 76, 147, 148]. Im Zweifelsfall sollte jede nicht durch eine innerhalb von 48 h vor dem Auftreten der Hyponatriämie verifizierte Normonatriämie als eine chronische Hyponatriämie betrachtet werden.

Die Geschwindigkeit, mit der die Hyponatriämie auftritt, ist deshalb von großer Bedeutung, weil das Gehirn in der Entstehung einer akuten Hyponatriämie noch nicht ausreichend Zeit gefunden hat, um entsprechende Kompensationsmechanismen (i. e. Shift osmotisch aktiver Substanzen wie organische Osmolyte und Elektrolyte von intra- nach extrazellulär; Wasserfluss aus dem Hirnparenchym in den Liquor) ausreichend umzusetzen [149, 150]. Aus diesem Grund gehen akute Hyponatriämien häufiger mit einer ausgeprägteren klinischen Symptomatik einher und müssen rascher und aggressiver behandelt werden. Akute Hyponatriämien entstehen in der Regel bei neu aufgetretener ADH-Ausschüttung verschiedener Ursachen und gleichzeitiger Ingestion hypotoner Flüssigkeiten. Auch Wasserintoxikationen, bei denen die renalen Kompensationsmöglichkeiten überwältigt werden, sind eine wichtige Ursache für akute Hyponatriämien [13, 134, 147].

Bei chronischer Hyponatriämie entwickelt sich die Hyponatriämie langsam, das Gehirn kann sich deshalb besser adaptieren, und die Hirnschwellung ist im Vergleich zu den akuten Hyponatriämien geringer ausgeprägt [150]. Während eine rasche Korrektur der akuten Hyponatriämie eben wegen dieser fehlenden Gegenregulationsmaßnahmen keine Komplikationen nach sich zieht, kann sie bei chronischen Hyponatriämien zu einer osmotischen Demyelinisierung führen (für weitere Details s. entsprechenden Abschnitt). Aus diesem Grund sollte eine maximale Korrekturrate von 8 mmol/l/Tag nicht überschritten werden.

### Iso- und hypertone Hyponatriämien

Iso und hypertone Hyponatriämien (inklusive Pseudohyponatriämien) bedürfen keiner Therapie zur Korrektur des Na^+^, da kein Abfall der S‑Osmolalität und somit keine echte Hyponatriämie mit der Gefahr der Entwicklung eines Hirnödems bestehen [151]. Hier stehen eine Diagnostik zur Ursachenfindung sowie die Therapie der Grunderkrankung im Vordergrund. In Folge wird somit nur die Therapie der hypotonen Hyponatriämie beschrieben.

### Akuttherapie bei symptomatischer Hyponatriämie

#### Definition der symptomatischen Hyponatriämie

Nach Identifizierung des Vorliegens einer hypotonen Hyponatriämie ist es entscheidend festzustellen, ob die Hyponatriämie als symptomatisch einzuordnen ist [2, 31, 76]. Diesbezüglich ist es für den behandelnden Arzt:Ärztin notwendig zu erkennen, ob die vorliegenden Symptome ursächlich auf die Hyponatriämie und nicht auf eine andere zugrunde liegende Pathologie zurückzuführen sind (z. B. Nausea und Emesis im Rahmen einer symptomatischen Hyponatriämie vs. zeitgleich bestehendem Ileus) [152]. Es sind nur hypotone Hyponatriämien mit mittel- bis schwerer Symptomatik unmittelbar therapiebedürftig (Tab. 1; [2, 31, 76]). Der zeitliche Verlauf des Auftretens einer Hyponatriämie (akut vs. chronisch) ist für die Therapieindikation nicht relevant.

#### Algorithmus zur Therapie der symptomatischen Hyponatriämie

Der Therapiealgorithmus der symptomatischen Hyponatriämie ist in Abb. 3 dargestellt.

Hyponatriämien mit schwerer Symptomatik bedürfen immer einer Behandlung mit hypertoner Kochsalzlösung (NaCl 3 %; 1026 mOsmol/LH_2_O). Grundsätzlich wird empfohlen, dass in Krankenhäusern entsprechende Lösungen vorgefertigt in geeigneten Gebinden (z. B. 150 ml) zur Verfügung stehen, um ggf. eine rasche Behandlung einleiten zu können.

Nach Bestätigung des Vorliegens einer Hyponatriämie (z. B. durch Blutgasanalyse) und Ausschluss einer Hyperglykämie-induzierten Hyponatriämie sollte bei Vorliegen von schweren bzw. mittleren auf die Hyponatriämie zurückführbaren Symptomen eine unmittelbare intravenöse Gabe von 2 ml/kgKG (max. 150 ml) NaCl 3 % erfolgen [31, 153]. Die Gabe von NaCl 3 % sollte i.v. über einen Zeitraum von 10 min erfolgen [76]. Wir empfehlen eine Kontrolle des S‑Na^+^ und der Klinik innerhalb von 10–20 min nach Gabe des NaCl 3 % [31]. Obwohl das S‑Na^+^ unmittelbar nach der Infusion ansteigt, benötigt es diese Zeitspanne, damit sich auch die klinische Symptomatik bessern kann (Rückgang des Hirnödems).

Bei lebensbedrohlicher Symptomatik (Atemstillstand, generalisierter Krampfanfall) kann im Einzelfall NaCl 3 % als Bolus auch schneller als über 10 min verabreicht werden.

Im Falle des initialen Vorliegens von schweren, auf die Hyponatriämie zurückführbaren Symptomen empfehlen wir eine Wiederholung der Gabe von 2 ml/kgKG (max. 150 ml) NaCl 3 % bei einem Ansteigen des S‑Na^+^< 4–6 mmol/l und/oder fehlender Verbesserung der Symptomatik. Insgesamt soll die Gabe nicht öfter als 3‑mal erfolgen [76].

Bei Persistenz der Symptomatik trotz adäquaten Anstiegs des S‑Na^+^ um bis zu 6 mmol/l nach Gabe von NaCl 3 % ist nach alternativen Ursachen für das Zustandsbild der Patient:innen zu suchen.

#### Korrekturrate

Im Rahmen der Korrektur einer Hyponatriämie stehen die Verbesserung einer etwaigen Symptomatik und die gleichzeitige Vermeidung einer Überkorrektur und damit verbundener Komplikationen (i. e. Auftreten eines osmotischen Demyelinisierungssyndroms [ODS]) im Vordergrund.

##### Kurzfristiges Therapieziel bei symptomatischer Hyponatriämie

Im Falle des Vorliegens einer symptomatischen Hyponatriämie mit mittlerer bzw. schwerer Symptomatik (s. Tab. 1) empfehlen wir eine akute Anhebung des S‑Na^+^ um 4–6 mmol/l [31, 76]. Ein Anstieg dieses Ausmaßes erscheint ausreichend, einer Erhöhung des Hirndrucks erfolgreich entgegenzuwirken (Reduktion um ca. 50 %) und zu einer Besserung des Zustandsbildes zu führen [154]. Bei schwerer Symptomatik sollte die Anhebung innerhalb von 1 h erfolgen [31, 76].

##### Generelle Empfehlungen zur Korrekturrate einer chronischen Hyponatriämie

Das Ziel ist, das S‑Na^+^ um 6–8 mmol/l/über jeden 24-h-Zeitraum anzuheben. Das zweite Ziel ist die Vermeidung einer korrekturassoziierten Komplikation im Sinne eines ODS. Die Inzidenz eines ODS ist in den Studien, welche eine Überkorrektur als > 10 mmol/l/in den ersten 24 h definierten, deutlich höher als in jenen Studien mit einem Cut-off von > 8 mmol/l [155, 156]. Auf Basis dieser Datenlage empfehlen wir eine Korrekturrate des S‑Na^+^ von maximal 8 mmol/l *über jeden 24-h‑Zeitraum* (= max. 16 mmol/l innerhalb von 48 h). Für Patient:innen mit Risikofaktoren für die Entwicklung eines ODS sollen niedrigere maximale Korrekturraten von 4–6 mmol/l über jeden 24-h-Zeitraum in Betracht gezogen werden [76, 157].

Neue, aber retrospektive Analysen deuten auf ein erhöhtes Mortalitätsrisiko bei niedriger Korrekturrate innerhalb der ersten 24 h hin (< 6–8 mmol/l/24 h) [12, 158]. Zusätzlich konnte in dieser Analyse keine eindeutige Assoziation zwischen der Korrekturrate in den ersten 24 h und dem Auftreten eines ODS gefunden werden [12, 159]. Diese Studien haben erhebliche Limitation: z. B. werde hauptsächlich nur die Natriumkorrekturrate innerhalb der ersten 24 h analysiert, und auf die Bedeutung der Grunderkrankung für die Mortalität wird nicht ausreichend eingegangen [160]. In Übereinstimmung mit einem Kommentar von Sterns et al. empfehlen wir, weiterhin die oben angeführten Zielvorgaben zur Korrektur der Hyponatriämie einzuhalten, da die oben genannte Studie keine ausreichende Evidenz liefert, eine raschere Korrekturrate zu empfehlen [160].

Formeln zur Berechnung der prognostizierten Änderung des S‑Na^+^ nach Gabe einer definierten Infusionslösung und -menge können aufgrund der gezeigten Ungenauigkeit auf Ebene der individuellen Patient:innen nicht empfohlen werden und ersetzen unter keinen Umständen eine engmaschige Kontrolle des Natriums [161–163].

##### Generelle Empfehlung zur Korrekturrate bei akuter Hyponatriämie

Für die akute Hyponatriämie muss keine maximale Korrekturrate in den ersten 24–48 h eingehalten werden [31]. Entsprechend den europäischen Empfehlungen kann bei nachweislichem akutem Abfall des S‑Na^+^> 10 mmol/l innerhalb von < 48 h wegen der Gefahr der Entwicklung einer akuten Symptomatik auch bei asymptomatischen Patienten ein Bolus von NaCl 3 % infundiert werden [31].

#### Korrekturmodalitäten: Bolus vs. kontinuierliche Gabe

In einer prospektiven, randomisierten, kontrollierten Studie mit 178 Patient:innen konnte gezeigt werden, dass eine Bolusgabe im Vergleich zu einer kontinuierlichen Gabe zu einem rascheren Anstieg des S‑Na^+^ führte bei gleicher Rate an Überkorrekturen (definiert als Anstieg des Serum-Natriums > 12/24 h bzw. 18 mmol/l/48 h) [164]. Die Notwendigkeit zur erneuten Absenkung des S‑Na^+^ aufgrund einer stattgehabten Überkorrektur war in der Bolusgruppe signifikant niedriger als in der kontinuierlichen Korrekturgruppe [164]. Auf Basis dieser Datenlage bevorzugen wir die Korrektur einer akuten bzw. einer symptomatischen, chronischen Hyponatriämie mittels Gabe eines Bolus NaCl 3 % gegenüber der prolongierten Gabe über einen Perfusor.

Wir empfehlen eine Dosis von 2 ml/kgKG mit einem Maximum von 150 ml für die Bolusgabe von NaCl 3 % zur raschen Korrektur einer akuten bzw. symptomatischen, chronischen Hyponatriämie [31]. Sollte das Körpergewicht unbekannt und nicht seriös zu schätzen sein, empfehlen wir eine Dosis von 100 ml NaCl 3 % als Bolusgabe [164].

#### Kontrollabstände des S-Natriums

Im Rahmen der raschen Korrektur einer akuten bzw. symptomatischen, chronischen Hyponatriämie empfehlen wir eine Kontrolle des S‑Na^+^ in einem Zeitraum von 10–20 min jeweils nach abgeschlossener Bolusgabe [31].

In jedem Fall empfehlen wir bei symptomatischen Patient:innen mit einer Hyponatriämie < 130 mmol/l innerhalb der ersten 24 h eine engmaschige Kontrolle des Natriumwerts (etwa alle 6 h), bis das S‑Na^+^ > 135 mmol/l ist [31]. Danach sollte zumindest 24 h später nochmals eine S‑Na^+^-Kontrolle erfolgen.

Bei Vorliegen von Risikofaktoren für ein zu rasches Ansteigen des Natriums und damit verbundener Überkorrektur empfehlen wir initial eine engmaschigere Kontrolle des Natriums (alle 2–4 h), um einer Überkorrektur frühzeitig vorbeugen zu können. Eine Übersicht über etablierte Risikofaktoren für eine Überkorrektur liefert Tab. 5.

**Tab. 5:** Risikofaktoren

Für eine Überkorrektur des S‑Na^+^	Für ein ODS
Hypokaliämie (< 3 mmol/l) Initial: S‑Na^+^ < 110 mmol/l Harnosmolalität < 150 mOsmol/kgH_2_O Erbrechen, Somnolenz *Weitere mögliche Faktoren* Weibliches Geschlecht Niedriger BMI Polydipsie, hohe Urinausscheidung Initiale Hypovolämie (H-Na^+^< 30 mmol/l) „Low solute intake“ Cortisolmangel Thiazideinnahme	Hyponatriämie > 48 h S‑Na^+^-Anstieg > 10–12 mmol/l/24 h Schwere Hyponatriämie (S-Na^+^ < 105 mmol/l) Schwere Hyponatriämie-assoziierte Symptomatik Malnutrition Chronischer Alkoholismus, Lebererkrankung Maligne Grunderkrankung Hypokaliämie Hypophosphatämie

Eine gleichzeitige Gabe von Kalium und/oder das Vorliegen einer Hypokaliämie sind mit einem rascheren Anstieg des Natriums assoziiert und müssen somit zwingend bei der Festlegung des Kontrollintervalls beachtet werden [30, 165].

Zur Verlaufskontrolle sollte die Natriumkonzentration immer mit derselben Methode (Serum bzw. Blutgasanalyse aus Vollblut venös oder arteriell) erfolgen, um rein verfahrenstechnische Schwankungen des Wertes zwischen den Kontrollen zu vermeiden [166–168]. Primär empfehlen wir die Kontrolle des S‑Na^+^ im Labor, wobei in der Akuttherapie bei schwerer Symptomatik aufgrund der raschen Verfügbarkeit oft die Messung im Blutgasanalysegerät durchgeführt werden muss.

Einer der Hauptgründe für einen zu raschen Anstieg des S‑Na^+^ (und damit verbunden einer Überkorrektur) ist eine hohe Urinausscheidung, weshalb wir eine Überwachung des ausgeschiedenen Urinvolumens empfehlen [169, 170]. Diesbezüglich wurde ein oberes Limit einer „sicheren“ Urinausscheidung von 1 ml/kgKG/h bzw. 24 ml/kgKG/24 h vorgeschlagen, wobei diese Empfehlung nicht extern validiert wurde [169].

Im Rahmen der Bilanzierung sollte auch das Körpergewicht am Aufnahmetag und im Verlauf dokumentiert werden. In vielen Fällen wird für eine derartig engmaschige Betreuung eine intensivmedizinische Überwachung erforderlich sein.

Bei leichtgradig symptomatischer (milder) Hyponatriämie sollte das S‑Na^+^ zumindest innerhalb von 24 h kontrolliert werden.

#### Überkorrektur der Hyponatriämie

##### Definition der Überkorrektur in der Behandlung einer Hyponatriämie

**Abb. 4:**
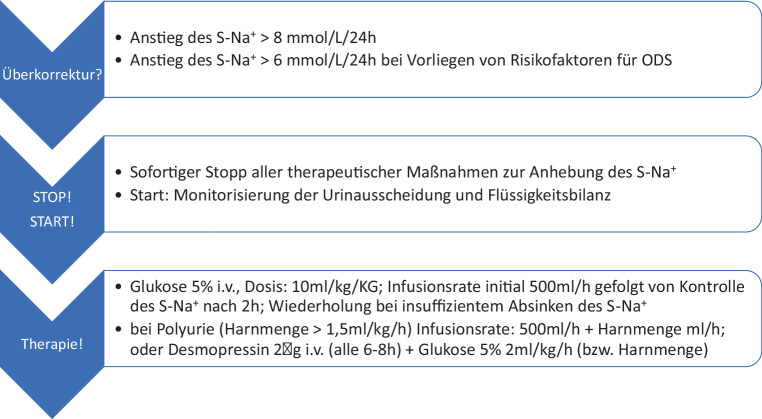
Maßnahmen bei Überkorrektur der Hyponatriämie

Neben den oben empfohlenen Zielen zur Korrekturrate des Natriums sollten auch die Limits beachtet werden, d. h. welche S‑Na^+^-Korrekturraten nicht überschritten werden dürfen, um ein ODS möglichst zu vermeiden. Eine Überkorrektur ist definiert als ein Anstieg des Serum-Natriums > 8 mmol/l/24 h. Bei Patient:innen mit Risikofaktoren für die Entwicklung eines ODS kann bereits ein Anstieg des Serum-Natriums > 6 mmol/l/24 h als Überkorrektur angesehen werden (Abb. 4).

##### Risikofaktoren für Überkorrektur einer Hyponatriämie

Die Risikofaktoren für eine Überkorrektur des Natriums sind in Tab. 5 abgebildet [31, 76, 165, 171–174]. Vor allem der rasche Wegfall des ADH-Stimulus, welcher sich durch eine niedrige Harnosmolalität und Polyurie äußert, führt zu einem raschen Anstieg des S‑Na^+^, z. B. nach Volumensubstitution bei reduziertem EABV durch Diarrhö oder nach Behandlung einer Nebenniereninsuffizienz mit Glukokortikoiden.

##### Therapeutische Maßnahmen bei Überkorrektur

Im Falle einer nachgewiesenen Überkorrektur sollten die Maßnahmen zur Anhebung des S‑Na^+^ umgehend gestoppt werden und folgende Maßnahmen eingeleitet werden *(Rescue-Therapie)*:

Als erste Maßnahme sollte zur neuerlichen Absenkung des S‑Na^+^ eine Infusion mit Glukose 5 % erfolgen. Glukose 5 % wird im Körper vollständig metabolisiert und entspricht somit der Zufuhr von freiem Wasser (ohne Elektrolyte); auch durch Trinken wird freies Wasser zugeführt. Es benötigt in etwa 3 ml/kgKG Glukose 5 % um das S‑Na^+^ um 1 mmol/l zu senken [64]. Deshalb muss in etwa eine Gesamtdosis von 10 ml/kgKG infundiert werden [31]. Eine Infusionsrate der Glukose 5 % von 500 ml/h (oder 5 ml/kg/h) wird bei Therapiebeginn empfohlen. Bei Polyurie muss die infundierte Menge an die Harnmenge (= 500 ml/h + Harnmenge/h) angepasst werden, um einen entsprechenden Abfall des S‑Na^+^ zu erzielen. Überschreitet die Infusionsrate > 500 ml/h, besteht das Risiko einer Glukosurie (bei Diabetes mellitus auch bei geringer Infusionsraten), welche aufgrund des Wasserverlustes durch osmotische Diurese einen Anstieg des S‑Na^+^ verursachen kann. In diesem Fall müsste auf eine Therapie mit Desmopressin in Kombination mit einer niedrigeren Infusionsrate von Glukose 5 % umgestellt werden (s. unten).

Unter der Therapie mit Glukose 5 % i.v. muss alle 1–2 h nach Infusionsbeginn das S‑Na^+^ kontrolliert werden. Im Falle eines nichtsuffizienten Abfalls in den Bereich des Zielkorrekturlimits empfehlen wir eine neuerliche Gabe (s. Abb. 4). Eine Monitorisierung der Urinausscheidung und der Flüssigkeitsbilanz ist hierbei zwingend nötig. Nachdem Patient:innen mit hohem Risiko für Überkorrektur bzw. ODS aufgrund ihrer Komorbiditäten (Tab. 5) auch für das Auftreten einer Wernicke-Enzephalopathie vulnerabel sind, sollte parallel zur Glukosegabe intravenös Thiamin verabreicht werden (wie weiter unten ausgeführt).

Alternativ kann bei Polyurie (Harnmenge > 1,5 ml/kg/h) Desmopressin 2 μg i.v. (z. B. Minirin® parenteral 4 µg/ml) verabreicht werden. Aufgrund der Wirkdauer muss die i.v.-Gabe je nach S‑Na^+^ in einem Intervall von 6–8 h wiederholt werden [31]. Auf eine intranasale Applikation von Desmopressin sollte wegen der schlechten Vorhersagbarkeit der Wirkung verzichtet werden. Auf diese Weise wird die Ausscheidung von elektrolytfreiem Wasser reduziert und so das S‑Na^+^ wieder abgesenkt, jedoch nur, wenn gleichzeitig Wasser zugeführt wird. Um das S‑Na^+^ effektiv abzusenken, muss daher parallel zu Desmopressin auch Glukose 5 % (Start: ca. 2 ml/kg/h) infundiert werden oder entsprechend viel getrunken werden (s. Abb. 4). Die Zufuhr an elektrolytfreiem Wasser (Infusionsrate von Glukose 5 %) sollte in Folge idealerweise an die Harnmenge angepasst werden. Bei vorheriger Therapie mit Tolvaptan ist die Therapie mit Desmopressin ineffektiv.

In einer Studie wurde auch der Einsatz von Desmopressin untersucht, wenn sich ein inadäquater schneller Anstieg des S‑Na^+^ abzeichnete *(reaktive Strategie)*. Dabei konnte gezeigt werden, dass die alleinige Gabe von Desmopressin den Anstieg des S‑Na^+^ reduziert, aber nur die gleichzeitige Gabe von freiem Wasser (Glukose 5 % i.v. oder Trinken) ein Absinken des S‑Na^+^ induziert [175–177].

##### Prävention der Überkorrektur

**Abb. 5:**
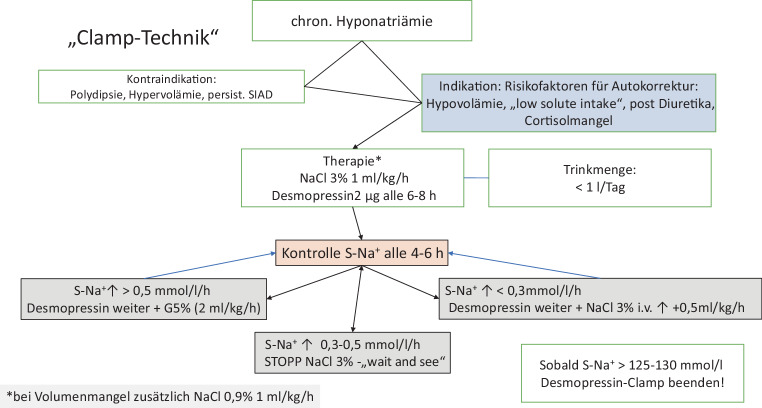
„Clamp-Technik“

Engmaschige Kontrollen des S‑Na^+^, Einhaltung der Dosierungsvorgaben von NaCl 3 % i.v. und Erkennen der Risikofaktoren (Tab. 5) für einen raschen Abfall des ADH-Spiegels sind die Basis für die Vermeidung einer Überkorrektur. Zudem kann der proaktive Einsatz von Desmopressin i.v. (2 µg alle 6–8 h) in Kombination mit NaCl 3 % (sog. „Clamp-Technik“, Abb. 5) helfen, eine Überkorrektur zu vermeiden [178–180]. Diese sollte nur in Situationen angewendet werden, in denen gleichzeitig ein hohes Risiko für eine Autokorrektur der Hyponatriämie (z. B. „low solute intake“, Hypovolämie) und das Auftreten eines ODS besteht (Abb. 5; [161]). Eine Polydipsie, ein SIAD und eine Hypervolämie sind Kontraindikationen für diese Behandlung. Wegen der Notwendigkeit eines entsprechenden Monitorings empfiehlt sich die Durchführung eines Desmopressin-„Clamps“ auf einer Überwachungsstation.

### Osmotisches Demyelinisierungssyndrom (ODS)

Das ODS beschreibt die neurologischen Folgen einer inadäquat schnellen Korrektur einer Hyponatriämie. Es wurde erstmals in den 1950er-Jahren bei Patient:innen mit Alkoholabhängigkeit und Mangelernährung beschrieben [181]. Der ursprüngliche Name zentrale pontine Myelinolyse wurde aufgrund zahlreicher Entdeckungen extrapontiner Manifestationen in ODS geändert. Obgleich äußerst selten, stellt das ODS die gefürchtetste Komplikation in der Behandlung einer Hyponatriämie dar.

#### Epidemiologie

Die Inzidenz eines ODS wird, abhängig von der untersuchten Fallserie, *bei Überkorrektur* des Natriums mit < 1–23 % angegeben, wobei die Definition einer Überkorrektur in den Studien zwischen > 8 mmol/l/24 h und > 25 mmol/l/48 h angegeben wurde [155, 156, 159, 182–187]. Neuere Studien zeigten eine Inzidenz von 0,05–0,3 % bei Betrachtung von Patienten mit einem S‑Natrium von < 130 bzw. 120 mmol/l, unabhängig von der Korrekturrate [12, 159, 160]. Durch die Einführung der Magnetresonanztomographie in der Diagnostik kam es zu einer Zunahme der Inzidenz des Krankheitsbildes [188].

#### Ätiologie

Malnutrition und/oder chronischer Alkoholabusus sind wichtige Risikofaktoren [157, 188]. Rezent wurden Hypokaliämie und Hypophosphatämie als weitere Risikofaktoren für die Entwicklung eines ODS identifiziert [157]. Die Hyponatriämie stellt aber den mit Abstand häufigsten ursächlichen Faktor für die Entstehung eines ODS dar [156, 189]. In einem systematischen Review konnte festgestellt werden, dass ca. 50 % aller Patient:innen mit ODS vorab eine schwere Hyponatriämie (definiert als ein S‑Na^+^ < 120 mmol/l) aufwiesen [190]. Laut aktuellen Richtlinien ist auch eine Korrekturrate von > 10–12 mmol/l/24 h mit einer höheren Rate an ODS assoziiert [31, 76]. Die Risikofaktoren für eine Überkorrektur der Hyponatriämie überlappen sich somit mit den Risikofaktoren für die Entwicklung eines ODS (s. Tab. 5). Es gibt jedoch auch Fälle von ODS trotz Einhaltung der Korrekturrate [191].

#### Pathogenese

Bei einer schweren, chronischen Hyponatriämie konnten im zentralen Nervensystem bereits Kompensationsmechanismen einsetzen, um das Zellvolumen auszugleichen (i. e. Shift von Elektrolyten und organischen Osmolyten aus der Zelle, s. Abschnitt akute Hyponatriämie) [149, 150]. Durch den zu raschen Anstieg der extrazellulären Osmolalität können die neuerlich notwendigen gegenläufigen Kompensationsmechanismen (Aufbau von intrazellulären Osmolyten) nicht vollständig einsetzen. Es shiftet Wasser aus der Zelle in den Extrazellularraum, und es kommt zu einer Abnahme des Zellvolumens mit daraus folgender Schädigung v. a. der Oligodendrozyten [192].

Die für ein ODS charakteristischen Läsionen werden als non-inflammatorische Demyelinisierungen, typischerweise symmetrisch auftretend, beschrieben, wobei Neurone und assoziierte Axone erhalten bleiben [188]. Außerdem kommt es zu einem Verlust von Oligodendrozyten sowie einer relevanten Infiltration mit Myelin-degradierenden Makrophagen [188, 192, 193].

#### Klinische Manifestation und Verlauf

Klinische Symptome treten in der Regel zwischen 1 und 14 Tagen nach dem auslösenden Ereignis (Überkorrektur einer meist profunden, chronischen Hyponatriämie) auf [188]. Die klinische Symptomatik eines ODS variiert sehr stark in Abhängigkeit von der Lokalisation der entstandenen Läsionen im ZNS. Die häufigsten Symptome sind dabei unspezifische Enzephalopathien, Vigilanzstörungen, Delir, Antriebslosigkeit, Gedächtnisstörungen und eine Beeinträchtigung der Konzentration [188, 194, 195]. Extrapontine Manifestationen resultieren häufig in Bewegungsstörungen wie Dystonie, Myoklonus, Rigor, Akinesie oder Tremor [188]. Anzumerken ist, dass der klinische Verlauf des ODS variiert; in 25 bis zu 39 % der Fälle verschwinden die Symptome vollständig. Im Gegensatz dazu benötigen 33–55 % der Betroffenen dauerhaft pflegerische Unterstützung nach der Erkrankung [188].

#### Diagnostik

Das Vorliegen eines klinischen Verdachts gepaart mit der Anamnese (Korrektur einer profunden, chronischen Hyponatriämie) sollte zur Durchführung einer Magnetresonanztomographie des Gehirns veranlassen. Die MRT weist eine höhere Sensitivität als eine CT-Untersuchung auf. Typische Läsionen zeigen sich hier als Hyperintensitäten in der T2-Wichtung sowie in den FLAIR Sequenzen [196]. Ein frühes MRT kann noch unauffällig sein (bis 4 Wochen nach Krankheitsmanifestation beschrieben), sodass bei anhaltendem klinischem Verdacht eine Verlaufskontrolle empfehlenswert ist [197].

#### Therapie

Im Vordergrund steht die Vermeidung des Auftretens eines ODS durch Einhaltung der empfohlenen Korrekturraten einer Hyponatriämie, v. a. bei Vorliegen von Risikofaktoren (s. Abschnitt Korrekturrate) [76].

Die im Rahmen eines ODS zur Verfügung stehenden Therapien basieren Großteils auf anekdotischen Berichten bzw. kleineren Fallserien, was den Evidenzgrad stark limitiert. Bei frühen Symptomen nach einer Überkorrektur sollte das S‑Na^+^ noch mal in den empfohlenen Korrekturbereich gesenkt werden, zusätzlich werden Glukokortikoide empfohlen; im Regelfall sind nur supportive Maßnahmen möglich [64, 76, 198]. Bei Mangelernährung, insbesondere bei Alkoholkranken, sollte großzügig Thiamin (Vitamin B_1_) substituiert werden (zumindest 100 mg i.v. pro Tag), da häufig zusätzlich ein Thiamin-Mangel besteht, welcher zu einer Wernicke-Enzephalopathie und vermutlich auch zu einem ODS führen kann [199].

### Therapie spezieller Formen der akuten Hyponatriämie

#### Therapie der Exercise-associated-Hyponatriämie (EAH)

Zum Vermeiden einer EAH sollte Trinken nach Maßgabe des Durstgefühls empfohlen werden [93]. Ebenso sollten bei entsprechenden Sportveranstaltungen salzhaltige Speisen und Getränke für die Teilnehmer zur Verfügung stehen.

Schwere Fälle einer EAH sollten, wie jede symptomatische Hyponatriämie, mittels intravenöser Gabe von NaCl 3 % behandelt werden [92, 93]. Bei gleichzeitigem Vorliegen eines Hitzschlags sind Kühlmaßnahmen empfohlen. Ist die EAH mild und präsentiert sich ohne neurologische Symptomatik, steht die Vermeidung von weiterem Trinken hypotoner Flüssigkeiten im Vordergrund [93]. Zudem kann eine orale Gabe hypertoner Flüssigkeiten durchgeführt werden. Eine konzentrierte Bouillon (4 Suppenwürfel gelöst in 125 ml Wasser) bzw. die orale Gabe von 100 ml NaCl 3 %-Lösung stellen hier eine therapeutische Möglichkeit dar [92]. Eine klinische Beobachtung und der Verzicht auf die Ingestion hypotoner Flüssigkeiten sollten bis zum Einsetzen einer Diurese beibehalten werden [92].

#### Therapie der leichtgradig symptomatischen akuten Hyponatriämie

Bei Nachweis einer akuten, leichtgradig symptomatischen Hyponatriämie mit einem Abfall des S‑Na^+^ um mehr als 10 mmol/l (innerhalb von < 48 h) sollte ebenfalls NaCl 3 % (2 ml/kg oder max. 150 ml) über 10 min infundiert werden. Das primäre Ziel ist, ein weiteres Absinken des S‑Na^+^ (durch Wasserresorption aus dem Gastrointestinaltrakt) zu verhindern und das S‑Na^+^ auf > 130 mmol/l anzuheben. Es besteht kein Risiko für ein ODS [31, 200].

### Therapie der chronischen und leichtgradig symptomatischen Hyponatriämie

Bei allen Patient:innen mit Hyponatriämie sollte eine kausale Therapie eingeleitet werden. In vielen Fällen (Hypovolämie, transiente ADH-Stimulation, Medikamente …) kann die Ursache der Hyponatriämie rasch behandelt werden, was aber auch die Gefahr einer schnell einsetzenden Aquarese und damit einen zu raschen Anstieg des S‑Na^+^ birgt. Es sollten immer die oben angeführten Korrekturraten eingehalten werden.

Bei der Therapieentscheidung muss auch überlegt werden, ob und wie lange die Therapie nach Korrektur der Hyponatriämie fortgesetzt werden muss; des Weiteren, wie oft Nachkontrollen notwendig sind. Dies ist primär davon abhängig, ob die Ursache erfolgreich therapiert werden konnte.

Für die meisten Therapiemöglichkeiten bei Hyponatriämie existieren keine randomisierten kontrollierten bzw. Langzeitstudien.

#### Therapie der chronischen Hyponatriämie nach Volumenstatus (EABV und EZV)

##### Hypovolämie

Die Ursachen für die Hypovolämie sollten behandelt werden (z. B. Absetzen von Diuretika). Das Grundprinzip besteht darin, die Barorezeptor-vermittelte ADH-Sekretion zu hemmen, indem der Volumenmangel durch eine i.v.-Therapie mit isotoner Flüssigkeit (Startdosis: 1–1,25 ml/kg/h) ausgeglichen wird [1, 201]. Es besteht ein erhöhtes Risiko eines inadäquat raschen Anstiegs des S‑Na^+^, da es nach Ausgleich der Hypovolämie zu einer Aquarese kommt. Deshalb sind engmaschige Kontrollen des S‑Na^+^ (alle 4–6 h) in dieser Situation erforderlich. Steigt die Harnmenge auf > 100 ml/h (oder 1 ml/kg/h) an, sollte kurzfristiger (alle 2–3 h) das S‑Na^+^ kontrolliert werden. Bei Hypovolämie kann auch in Einzelfällen (zuvor Behandlung der symptomatischen Hyponatriämie mit NaCl 3 %-Bolus) die proaktive Desmopressin-Therapie oder auch „Clamp-Technik“ überlegt werden. Zu beachten ist, dass bisher nur Proof-of-concept-Studien dazu veröffentlicht wurden (s. Abb. 5 und Abschnitt Überkorrektur) [161, 179, 180].

##### Hypervolämie

Die Herausforderung besteht in der parallelen Behandlung der Hypervolämie (Induktion einer negativen Natriumbilanz, da die Natriummenge das Extrazellularvolumen definiert) und der durch die Hyponatriämie bedingten Hypoosmolalität (Induktion einer Aquarese).

- Herzinsuffizienz

Eine Normalisierung des S‑Na^+^ bei akuter dekompensierter Herzinsuffizienz ist mit einem besseren Überleben assoziiert als bei persistierender Hyponatriämie [202]. Eine Flüssigkeitsrestriktion ist empfehlenswert (< 1–1,5 l/Tag, neben Trinkmenge auch Suppen etc. beachten), ist aber aufgrund des starken Durstgefühls bei vielen Patient:innen nicht immer umsetzbar [203]. Um eine Dekongestion zu induzieren, werden zusätzlich zur Flüssigkeitsrestriktion Diuretika benötigt. Die Therapie mit Schleifendiuretika ist das Mittel der Wahl in dieser Konstellation. Schleifendiuretika stören den Aufbau des Konzentrationsgradienten in der Niere und schwächen damit die Wirkung von ADH ab – die Folge ist eine Verbesserung der Natriurese und Aquarese und ein Anstieg des S‑Na^+^ [204].

Der alleinige Einsatz von Tolvaptan (selektiver Vasopressin-2-Rezeptor-Antagonist) in der Therapie der Hyponatriämie bei Herzinsuffizienz kann derzeit generell weder in der Phase der akuten Dekompensation noch in der Langzeittherapie empfohlen werden. Obwohl in entsprechenden Studien ein Trend zur Besserung der Hyponatriämie beobachtet wurde, gab es keine positiven Effekte auf Langzeitergebnisse wie Mortalität [205–207].

Ob sich der zunehmende Einsatz von SGLT2-Hemmer positiv auf die S‑Natriumwerte auswirkt, ist aktuell noch nicht ausreichend beurteilbar. Durch die Induktion einer osmotischen Diurese sollte die EFWC erhöht werden und damit das S‑Na^+^ angehoben werden [208].

Die Kombination von NaCl 3 % i.v. mit Furosemid, welche als Rescue-Therapie bei therapierefraktärer kardialer Dekompensation eingesetzt wird, kann als Therapie der Hyponatriämie zwar nicht empfohlen werden, führt aber wahrscheinlich nicht zu einer erhöhten Rate an Überkorrekturen [209].

- Lebererkrankungen

Eine Flüssigkeitsrestriktion von max. 1–1,5 l/Tag wird empfohlen, führt aber nur bei etwa einem Drittel der Patient:innen zu einem Anstieg des S‑Na^+^ um > 5 mmol/l innerhalb von 2 bis 3 Tagen [210]. Die i.v.-Substitution von Albumin ist hingegen deutlich effektiver [211, 212]. Vasopressin-2-Rezeptor-Antagonisten wurden v. a. bei Leberzirrhose mit Aszites und chronischer Hyponatriämie eingesetzt und führten ebenfalls zu einem Anstieg des S‑Na^+^ [213]. Zumindest in einer Studie konnte eine Assoziation zwischen der Normalisierung des S‑Na^+^ unter Tolvaptan und einer Verbesserung der 6‑Monats-Überlebensrate beobachtet werden [143]. In Abhängigkeit der Aktivität von ADH und des Ausmaßes der Hyponatriämie kann die Therapie mit Tolvaptan aber auch ineffektiv sein [214, 215]. Die FDA warnt aufgrund der potenziellen Hepatotoxizität vor einer Langzeittherapie mit Tolvaptan bei Patienten mit Leberzirrhose, obwohl neuere Studien dies nicht bestätigen konnten [213, 216].

Das temporäre Absetzen von Diuretika, v. a. jener, welche die Harndilution stören (Thiazide, Aldosteronantagonisten), wird ebenfalls empfohlen [128]. Der Einsatz von Harnstoff kann im Kontext von Lebererkrankungen aufgrund der möglichen Erhöhung der Ammoniakproduktion nicht empfohlen werden [128].

Erste Daten zur Kombination von Terlipressin und Albumin bei der Behandlung des hepatorenalen Syndroms mit akutem Nierenversagen zeigten kein erhöhtes Risiko für das Auftreten von Hyponatriämien (CONFIRM-Studie) [131].

- Hyponatriämie bei Nierenerkrankungen

Hier empfiehlt sich der Einsatz von Schleifendiuretika (in entsprechend höherer Dosierung) in Kombination mit Flüssigkeitsrestriktion zur Therapie der Hypervolämie und Hyponatriämie [76]. Thiaziddiuretika sollten hingegen vermieden werden. In Einzelfällen kann der kurzfristige Einsatz von Tolvaptan (meist in Kombination mit Furosemid) bei therapierefraktären Ödemen und nephrotischem Syndrom erwogen werden [76, 217].

Terminales Nierenversagen: Bei schweren, symptomatischen Hyponatriämien und Nierenversagen wird ebenfalls NaCl 3 % in der Akuttherapie eingesetzt. Da oft gleichzeitig eine Hypervolämie besteht oder der Patient aus anderen Gründen eine Nierenersatztherapie benötigt, muss – um eine inadäquate Korrekturrate des S‑Na^+^ zu verhindern – das Dialysat-Natrium angepasst werden [218]. Dies kann durch kurze intermittierende Hämodialysebehandlungen (max. 3 h) mit minimalem Dialysat-Natrium (130 mmol/l) und niedrigem Blutfluss (100 ml/min) erreicht werden. Besser ist es jedoch, eine kontinuierliche Nierenersatztherapie (Hämofiltration) anzuwenden und das Dialysat-Natrium in den Lösungsbeutel durch Zugabe von Glukose 5 % der aktuellen S‑Na-Konzentration anzupassen (für Details s. Supplement 1 [218]).

##### Euvolämie

- Primäre Polydipsie

Die Flüssigkeitsrestriktion ist die wichtigste Maßnahme zur Therapie der Polydipsie, aber oft schwierig umzusetzen [67]. Das Ausmaß der notwendigen Flüssigkeitsrestriktion ist abhängig von der Nahrungszufuhr (s. „low solute intake“). Wird eine niedrigste Harnosmolalität von 100 mOsmol/kgH_2_O angenommen, so reicht bei einer normalen Zufuhr an osmotischen Substanzen (10 mOsmol/kg/Tag) eine Flüssigkeitsrestriktion von < 5 l/Tag aus, um das S‑Na^+^ zu korrigieren. Als symptomatische Therapie kann Harnstoff p.o. eingesetzt werden (0,3–0,9 g/kg/Tag), welcher die Zufuhr an osmotischen Substanzen erhöht und damit die renale Wassereliminationskapazität erhöht [219]. Der zentral wirksame GLP1-Agonist Dulaglutide hemmt das Durstgefühl, reduziert die Trinkmenge und erhöht somit ebenfalls das S‑Na^+^ [220]. Die Evidenz für die Behandlung mit Harnstoff bzw. Dulaglutide ist begrenzt, beide Therapieoptionen können aber als risikoarm eingestuft werden. Die Patient:innen benötigen langfristig eine psychiatrische Evaluierung und eine darauf basierende Therapie der Grundproblematik [66].

- „Low solute intake“

Bei dieser Ursache der Hyponatriämie besteht ein Missverhältnis zwischen Trinkmenge und Nahrungszufuhr (= osmotische Substanzen). Im Gegensatz zur primären Polydipsie sollte primär die Menge an osmotischen Substanzen in der Nahrung erhöht werden. Zur Korrektur der Hyponatriämie kann Harnstoff p.o. eingesetzt werden; 30 g Harnstoff entsprechen ca. 500 mOsmol osmotisch wirksamer Substanzen. Derselben Menge an osmotischen Substanzen entspricht die Zufuhr von 15 g NaCl oder 88 g Protein. Es reichen aber in diesem Fall meist 15–20 g Harnstoff aus, um die Hyponatriämie zu korrigieren. Auch eine Flüssigkeitsrestriktion von < 1 l/Tag kann effektiv das S‑Na^+^ anheben. Eine Evidenz, basierend auf randomisierten Studien, gibt es für diese Therapieansätze nicht.

#### SIAD (Syndrom der inadäquaten Antidiurese)

Bei einem SIAD sollte immer eine mögliche Ursache (s. Tab. 3) gesucht werden, um idealerweise kausal behandeln zu können (Absetzen auslösender Medikamente, Behandlung eines Malignoms etc.). Für das SIAD gibt es multiple Therapieansätze, welche einzeln oder in Kombination eingesetzt werden können. Das Problem in der Interpretation der Therapiestudien besteht darin, dass in keiner dieser Studien transiente SIADs ausgeschlossen werden konnten und so die Möglichkeit der spontanen Besserung bestand. Zusätzlich gilt es zu beachten, dass die Infusion von Plasma-isotonen Lösungen bei SIAD zu einem weiteren Abfall des S‑Na^+^ führen kann. Dies gilt für alle Situationen, bei denen die Harn-Na^+^K^+^-Konzentration > als die S‑Na^+^-Konzentration ist [76].

##### Flüssigkeitsrestriktion

Eine Flüssigkeitsrestriktion wird beim SIAD generell empfohlen [1, 76]. Das Ausmaß der Restriktion kann durch die Berechnung der Na^+^K^+^-Konzentration im Harn in Relation zur S‑Natrium-Konzentration abgeschätzt werden (Furst-Formel: Supplement 4) [221]. Eine Flüssigkeitsrestriktion von max. 1000 ml/Tag führt zu einer Anhebung des S‑Na^+^ um ca. 3–4 mmol/l bei jedoch nur ca. 50 % Therapieresponder [222–224]. In einer diesen Studien konnte neben einer Furst-Ratio > 1,0 auch eine Harnosmolalität > 500 mOsmol/kgH_2_O als prädiktiv für das Nichtansprechen auf eine alleinige Flüssigkeitsrestriktion gefunden werden [224]. In diesen Fall muss die Flüssigkeitsrestriktion mit einer Osmotherapie oder Tolvaptan kombiniert werden. Prinzipiell ist festzuhalten, dass eine dauerhafte „strenge“ Flüssigkeitsbeschränkung (z. B. ≤ 1 l/Tag) den Patient:innen nicht zumutbar ist, eine pragmatische Reduktion der Trinkmenge (etwa auf 1,5 l/Tag) jedoch auch als Teil anderer Therapieoptionen sinnvoll ist.

##### Osmotherapie mit Harnstoff oder Protein

Ein potenzieller Kombinationspartner für die Flüssigkeitsrestriktion ist die Therapie mit Harnstoff per os. Durch die Gabe von Zusatzstoffen kann die geschmackliche Akzeptanz erhöht werden (z. B. „*Brussels Champagne*“, s. Supplement 5) [225]. Harnstoff induziert eine osmotische Diurese und erhöht damit die Aquarese; 15 g Harnstoff entsprechen 250 mOsmol osmotischer Substanz und führen bei einer Harnosmolalität von 500 mOsmol/kgH_2_O zur Ausscheidung von maximal 500 ml elektrolytfreiem Wasser. Es wird die Einnahme von 0,25–0,5 g/kg Harnstoff pro Tag empfohlen, die Dosis kann je nach Wirkung bis auf max. 90 g/Tag erhöht werden, die Gabe sollte auf maximal 2 Einzeldosen aufgeteilt werden [76]. Auch Langzeitdaten für die Harnstofftherapie existieren, jedoch nicht in Form von randomisierten kontrollierten Studien [226–230]. Die Harnstofftherapie führt naturgemäß zu einem Anstieg der Harnstoffkonzentration im Blut (bis auf das Doppelte); dies ist aber kein Zeichen einer Verschlechterung der Nierenfunktion [231]. Die Therapie führt nur selten zu einem inadäquat raschen Anstieg des S‑Na^+^ und dürfte protektiv gegenüber der Entwicklung einer osmotischen Demyelinisierung sein [232]. Äquivalent zur Harnstofftherapie von 30 g (oder 0,25–0,5 g/kg) kann auch Proteinpulver mit ca. 90 g/Tag eingesetzt werden, wobei aber dazu keine Langzeitdaten existieren [233]. Die Osmotherapie mit Kochsalz wird nicht empfohlen, da die Äquivalenzdosis von 30 g Harnstoff 15 g Kochsalz entspricht. Vorgefertigte Harnstoffpräparate (Ure-Na®, UreaAide®) sind in Österreich noch nicht verfügbar, allerdings kann Harnstoff (bzw. *Brussels Champagne*) magistraliter verordnet werden, wobei die Hydrophilie der Substanz die Lagerung erschwert. Die typische Einzeldosis von 15 g entspricht dem Volumen eines Messlöffels für Resonium® oder 2 gestrichenen Teelöffeln.

##### Vasopressin-2-Rezeptor-Antagonisten

Bei fehlendem Therapieansprechen auf Flüssigkeitsrestriktion ± Osmotherapie sollte der orale Vasopressin-2-Rezeptor-Antagonist Tolvaptan eingesetzt werden [234, 235], welcher allerdings nicht immer relevante klinische Endpunkte verbessert, wie z. B. neurokognitive Funktionen [144, 234]. Aufgrund der möglichen raschen Anhebung des S‑Na^+^ und der eingeschränkten Möglichkeit zur Therapie der Überkorrektur mit Desmopressin (nur Glukose 5 % i.v. ist effektiv) sollte Tolvaptan primär nur bei S‑Na^+^ > 125 mmol/l eingeleitet werden. Die Dosis von Tolvaptan sollte mit 3,75–7,5 mg/Tag im stationären Bereich begonnen und unter engmaschiger Kontrolle des S‑Na^+^ langsam nach oben titriert werden (in Einzelfällen bis max. 90 mg/Tag) [236]. Unter Tolvaptan darf keine Flüssigkeitsrestriktion oder zusätzliche Kombinationstherapie (Harnstoff, Furosemid) durchgeführt werden, und die Patient:innen müssen Zugang zu Wasser haben [10]. Auch über eine längere Therapiedauer sind die Effektivität und Sicherheit untersucht worden [237]. Regelmäßige Kontrollen der Leberwerte unter Tolvaptan-Therapie sind notwendig. Bei Erhöhung der Transaminasen über das 2‑ bis 3fache des Normalwertes sollte die Therapie abgesetzt werden. Typische Nebenwirkungen sind vermehrtes Durstgefühl und Polyurie. Die Rate an Überkorrekturen in den einzelnen Studien variiert zwischen 0 und 30 % und ist stark abhängig von der verwendeten Dosierung bzw. vom Ausgangswert des S‑Na^+^ [10]. Kleine Packungsgrößen und hohe Therapiekosten erschweren eine Dauertherapie, wie sie unter manchen Umständen notwendig sein kann. Außerhalb der Zulassung wird vereinzelt auch eine intermittierende Gabe (z. B. jeden zweiten Tag) praktiziert.

##### SGLT2-Hemmer

Rezente Studien zeigen, dass auch Empagliflozin in Kombination mit Flüssigkeitsrestriktion durch die Induktion einer osmotischen Diurese das S‑Na^+^ steigern kann [238]. Dabei konnte in einer Studie auch eine Verbesserung der neurokognitiven Situation mit Empagliflozin ohne Flüssigkeitsrestriktion vs. Placebo beobachtet werden [239]. Zur effektiven Wirkung sollte die Dosis von Empagliflozin 25 mg mit einer Flüssigkeitsrestriktion von max. 1 l/Tag kombiniert werden. Die Therapie mit Empagliflozin wurde bis auf ein vermehrtes Durstgefühl gut vertragen; es wurden keine überschießenden Anstiege des S‑Na^+^ beobachtet – Langzeitdaten hinsichtlich Effektivität und Nebenwirkungen stehen jedoch noch aus.

##### Furosemid

Die Therapie mit Furosemid kann nicht für die Langzeittherapie bei SIAD empfohlen werden, da das Risiko für die Induktion einer Hypovolämie oder Hypokaliämie und Hypomagnesiämie erhöht ist und auch keine ausreichende Effektivität beschrieben wird [223].

##### Therapie bei Nebennierenrindeninsuffizienz-Hypocortisolismus

Bei nachgewiesenem oder bereits bei v. a. einen schweren Hypocortisolismus (Hyponatriämie, Hypotonie, Hypoglykämie) sollte sofort eine i.v.-Therapie mit Hydrocortison 100 mg Bolus, gefolgt von 50 mg alle 6 h i.v., begonnen werden [114]. Diese Therapie muss immer mit der Gabe von isotonen Kochsalzlösungen (ca. 1 ml/kg/h) kombiniert werden, da bei einer Nebenniereninsuffizienz von einer intravasalen Hypovolämie auszugehen ist. In Folge sollte auf das Einsetzen einer Aquarese geachtet werden, um einer Überkorrektur rechtzeitig entgegenwirken zu können. Bei primärer Nebennierenrindeninsuffizienz kann gleichzeitig auch eine Therapie mit Mineralkortikoiden (Fludrocortison 50–100 µg/Tag) eingeleitet werden [114].

## Umrechnungsfaktoren

Zur Berechnung der Osmolalität im Harn und Serum müssen die osmotisch aktiven Substanzen Harnstoff und Glukose, welche im Labor meist in mg/dl angegeben werden, in mmol/l umgerechnet werden:

- Glukose:
  - Glukose in mg/dl*0,0555 = Glukose in mmol/l (= mg/dl/18)
- Harnstoff und Harnstoff-Stickstoff (BUN):
  - Harnstoff in mg/dl*0,167 = Harnstoff in mmol/l (= Harnstoff in mg/dl/6,006)
  - Harnstoff-N(= BUN) in mg/dl*0,357 = Harnstoff‑N in mmol/l (= BUN in mg/dl/2,8)
  - Die Konzentration von Harnstoff und Harnstoff‑N in mmol/l ist ident!
- Cortisol:
  - Cortisol in µg/dl*27,58 = Cortisol in nmol/l

## Anhang

### Supplement 1: Berechnung des Substituat-Natriums bei CVVHF

Die Konzentration von Natrium im Substituat bei einer kontinuierlichen Hämofiltration muss an das S‑Natrium angepasst werden, um eine zu rasche Korrektur zu verhindern. Es wird die Berechnung der Natriumkonzentration im Substituat bei Verwendung einer CVVHF-Postdilution zur Therapie der Hyponatriämie beim Nierenversagen gezeigt [218]:

1. Berechnung der Na-Dialysance:

$$\mathrm{D}=\mathrm{SC}(\mathrm{Na})*(\mathrm{Q}(\mathrm{RF})+\mathrm{Q}(\mathrm{UF}))$$

SC(Na)...: Siebkoeffizient von Natrium = 1;
Q(RF)…: Fluss der „replacement fluid“ (= Substituat) in ml/h,
Quf…: Ultrafiltrationsrate in ml/h

2) Berechnung der notwendigen Natriumkonzentration im Substituat zum Erreichen des Ziel-Natriums:

$$\mathrm{Na}\left(\mathrm{RF}\right)=\text{S-Na}\left(\text{aktuell}\right)+\left(\frac{\text{Ziel}\Updelta \text{S-Na}}{1-e^{\frac{\left(-\mathrm{D}*24\right)}{\mathrm{V}}}}\right)$$

Na(RF)…: Na-Konzentration im Substituat in mmol/l,
D…: Dialysance,
V…: Gesamtkörperwasser in l (ca. 0,5 × KG(kg) für Frauen und 0,6 × KG(kg) für Männer)

3) Berechnung der Zugabe von Glukose 5 % in das Substituat, um die gewünschte Natriumkonzentration im Dialysat zu erhalten.

$$\begin{aligned}&\text{Zusatz}\,\mathrm{G}5{\%}=\\ &\mathrm{RF}\text{-Menge}*\left(\frac{\mathrm{Na}\,\text{initial}\left(\mathrm{RF}\right)-\mathrm{Na}\,\text{Ziel}\left(\mathrm{RF}\right)}{\mathrm{Na}\,\text{initial}\,\left(\mathrm{RF}\right)}\right)\end{aligned}$$

Zusatz G5%...: Menge an G5% (in l), welche dem Substitualbeutel hinzugefügt werden muss
RF-Menge: Flüssigkeitsmenge des Substituatbeutels (in l),
Na(RF): Natriumkonzentration im Substituat in mmol/l,
Na initial…: Na-Konzentration im Substituat vor der Zugabe des Zusatzes,
Na Ziel (RF): gewünschte Natriumkonzentration im Dialysat

### Supplement 2: Messung und Berechnung der Osmolalität im Serum und Harn

Die Osmolalität bezeichnet den Anteil an osmotischen Teilchen pro Kilogramm Lösungsmittel (z. B. Harn) und wird deshalb in mOsmol/kg angegeben. Die Osmolarität wird hingegen auf die Menge an Lösungsmittel bezogen und wird deshalb in mOsmol/l angegeben. Wird die S‑Osmolalität berechnet, so wird eigentlich die *S‑Osmolarität* berechnet, da die verwendeten Parameter alle in mmol/l angegeben werden [240]. Es muss deshalb dafür korrigiert werden.

*Berechnung der S‑Osmolalität* [241]:

$$\begin{aligned}&\mathrm{S}\text{-Osmolalit{\"a}t}_{\mathrm{calc}}\left(\frac{\text{mOsmol}}{\text{kg}}\right)=\\ &1{,}86x\text{S-Na}\,\left(\frac{\text{mmol}}{\text{l}}\right)+\frac{\text{S-BUN}\,\left(\frac{\text{mg}}{\text{dl}}\right)}{2{,}8}+\\ &\frac{\mathrm{S}\text{-Glukose}\,\left(\frac{\text{mg}}{\text{dl}}\right)}{18}+9\end{aligned}$$

Es ist eine Vielzahl von anderen Berechnungen der S‑Osmolalität publiziert [240]. Die Konzentrationen von BUN und Glukose müssen in mmol/l umgerechnet werden. Man beachte bei der Verwendung der Formel den Unterschied zwischen Harnstoff bzw. Harnstoff-Stickstoff (BUN) (s. Umrechnungsformeln). Bei Verwendung der Harnstoffkonzentration (statt BUN) muss durch 6,006 dividiert werden, um mg/dl in mmol/l umzuwandeln.

Die Differenz zwischen gemessener und berechneter S‑Osmolalität wird als Osmolalitätslücke bezeichnet.

*Osmolalitätslücke*:

$$\mathrm{S}\text{-Osmolalit{\"a}tsl{\"u}cke}=\mathrm{S}\text{-Osmo}_{\mathrm{gem}}-\mathrm{S}\text{-Osmolalit{\"a}t}_{\mathrm{ber}}$$

> 10 mOsmol/kgH_2_O ist pathologisch.

Bei einer Hyponatriämie ist eine Verminderung der gemessenen S‑Osmolalität zu erwarten. Die Osmolalitätslücke erklärt sich durch eine Akkumulation von nicht in der Kalkulation enthaltenen osmotischen Substanzen. Mögliche Konstellationen für eine Übereinstimmung oder Diskordanz zwischen berechneter und gemessener S‑Osmolalität sind in Tab. 6 angegeben [44].

**Tab. 6:** Hyponatriämie (S-Na^+^ < 135 mmol/l) +

S‑Osmolalität (gem.)	S‑Osmolalität (ber.)	[Na^+^] in BGA	Interpretation
> 275 mOsmol/kgH_2_O	> 275 mOsmol/kgH_2_O	< 135 mmol/l	Nierenversagen – BUN ↑ Glukose ↑
> 275 mOsmol/kgH_2_O	< 275 mOsmol/kgH_2_O^a^	< 135 mmol/l	Mannitol, Sorbitol, Röntgenkontrastmittel Ethanol, Methanol
> 275 mOsmol/kgH_2_O	< 275 mOsmol/kgH_2_O^a^	> 135 mmol/l	Pseudohyponatriämie (Chol ↑, TG ↑, Prot. ↑)

*BGA* Blutgasanalyse

^a^*Osmo-Gap >* *10* *mOsmol/kgH*_*2*_*O*

Ineffektive Osmolyte: BUN, Ethanol, Methanol = hypotone Hyponatriämie

Effektive Osmolyte: Glukose, Mannitol, Sorbitol, Röntgenkontrastmittel = translokationale Hyponatriämie

Die gemessene S‑Osmolalität kann für osmotisch ineffektive Faktoren korrigiert werden, um die effektive S‑Osmolalität zu erkennen.

*S‑Osmolalität Korrektur für BUN („effektive berechnete S‑Osmolalität“)*:

$$\begin{aligned}&\mathrm{BUN}\,\text{korrigierte}\,\mathrm{S}\text{-Osmolalit{\"a}t}=\\ &\mathrm{S}\text{-Osmo}_{\mathrm{gem}}-\frac{\mathrm{BUN}_{\mathrm{gem}}\,\left(\frac{\text{mg}}{\text{dl}}\right)}{2{,}8}\end{aligned}$$

Bsp. S‑Na 125 mmol/l, BUN 120 mg/dl, gemessen S‑Osmolalität 280 mOsmol/kgH_2_O, Blutzucker 100 mg/dl. BUN-korrigierte S‑Osmo = 280–43 = 237 mOsmol/kg. Es besteht eine hypotone Hyponatriämie.

*S‑Osmolalität Korrektur für Ethanol*:

$$\begin{aligned}&\text{Ethanol}\,\text{korrigierte}\,\mathrm{S}\text{-Osmolalit{\"a}t}=\\ &\mathrm{S}\text{-Osmo}_{\mathrm{gem}}-\frac{\text{Ethanol}_{\mathrm{gem}}\,\left(\frac{\text{mg}}{\text{dl}}\right)}{3{,}7}\end{aligned}$$

Andere osmotisch ineffektive Teilchen wie Methanol werden nicht berücksichtigt.

Die gemessene S‑Osmolalität kann auch für osmotisch effektive Teilchen, wie Glukose korrigiert werden. Es wird der Effekt der Glukosekonzentration auf die S‑Osmolalität bzw. S‑Na^+^ angezeigt.

*S‑Osmolalität Korrektur für Hyperglykämie*:

$$\begin{aligned}&\text{Glukose}\,\text{korrigierte}\,\mathrm{S}\text{-Osmolalit{\"a}t}=\\ &\mathrm{S}\text{-Osmo}_{\mathrm{gem}}-\frac{\mathrm{S}\text{-Glukose}_{\mathrm{gem}}\,\left(\frac{\text{mg}}{\text{dl}}\right)}{18}\end{aligned}$$

*S‑Na*^*+*^* Korrektur bei Hyperglykämie*:

$$\text{S-Na}_{\mathrm{corr}}=\text{S-Na}_{\mathrm{gem}}+2{,}4*\frac{\text{Glukose}_{\mathrm{gem}}\,\left(\frac{\text{mg}}{\text{dl}}\right)-100\,\left(\frac{\text{mg}}{\text{dl}}\right)}{100\,\left(\frac{\text{mg}}{\text{dl}}\right)}$$

*Harnosmolalität (s. *Gl. 2*)*:

Die Berechnung der Harnosmolalität nach *Gl.* 2 beruht, ähnlich wie bei der S‑Osmolalität, auf der Berechnung der *Osmolarität* [46]. Aus didaktischen Gründen werden beide Begriffe synonym verwendet. Die Berechnung der Harnosmolalität gelingt auch näherungsweise durch die Multiplikation der Zahlen hinter der Kommastelle der Harndichte mit dem Faktor 35 [242].

Beispiel: Harndichte 1,0***20*** = 20*35 = 700 mOsmol/kgH_2_O.

### Supplement 3: Berechnung der fraktionellen Exkretionsrate von Harnsäure

Die fraktionelle Exkretionsrate von Harnsäure (FeHS):

$$\begin{aligned}&\mathrm{FE}\text{-Harns{\"a}ure}=\\ &\frac{\mathrm{U}\text{-Harns{\"a}ure}\,\left(\frac{\text{mg}}{\text{dl}}\right)*\mathrm{S}\text{-Creatinine}\,\left(\frac{\text{mg}}{\text{dl}}\right)}{\mathrm{U}\text{-Creatinine}\,\left(\frac{\text{mg}}{\text{dl}}\right)*\mathrm{S}\text{-Harns{\"a}ure}\,\left(\frac{\text{mg}}{\text{dl}}\right)}*100\left({\%}\right)\end{aligned}$$

Die Berechnung der FeHS hilft in der Differenzialdiagnose der Hyponatriämie. Bei SIAD ist die FeHS > 12 %. Während bei anderen Ursachen der Hyponatriämie (mit verminderten EABV) die FeHS bei < 8 % liegt [77].

### Supplement 4: Furst-Ratio

Furst-Ratio [221]: Zur Evaluierung des Therapieerfolges einer Flüssigkeitsrestriktion bei SIAD.

- $\text{Furst-Ratio}=\frac{\mathrm{U}-\left(\text{Na+K}\right)}{\text{S-Na}}$ (alle in mmol/l)
- Furst-Ratio < 0,5: Flüssigkeitsrestriktion 1000 ml/Tag
- Furst-Ratio 0,5–1: Flüssigkeitsrestriktion 500 ml/Tag
- Furst-Ratio > 1: Flüssigkeitsrestriktion allein nicht ausreichend

### Supplement 5: Magistraliter-Rezeptur für oralen Harnstoff

„Brussels Champagne“ – Einzeldosis von 10 g Harnstoff [225]

- Urea/Carbamidum: 10 g
- Natriumhydrogencarbonat: 2 g
- Zitronensäure: 1,5 g
- Saccharose: 200 mg^1^
- Gelöst: in 50–100 ml Wasser

Dosiseffekt von Harnstoff auf die renale Wasserausscheidung

$$\text{max.EFWC}\,\left(\text{ml}\right)=\frac{\text{Harnstoff\,p.o.}\,\left(\text{mOsmol}\right)}{\left(\text{minimale}\right)\mathrm{H}\text{-Osmol}\,\left(\frac{\text{mOsmol}}{\text{kgH2O}}\right)}$$

Bsp.: H‑Osmolalität 600 mOsmol/kgH_2_O, Harnstoff 2 × 15 g/Tag (= 500 mOsmol). Maximale Menge an (elektrolytfreiem) Wasser, das *zusätzlich* ausgeschieden werden kann: ca. 830 ml.

### Supplement 6: Wasserbelastungstest

Bei unklaren Fällen eines milden SIAD (SNa^+^ > 130 mmol/l) und zur Diagnose eines Reset-Osmostat kann ein Wasserbelastungstest durchgeführt werden. Der Patient muss dabei 20 ml/kg Wasser innerhalb von 15–30 min trinken. Alternativ kann in etwa dieselbe Menge an Glukose 5 % i.v. infundiert werden [243, 244].

Normalbefund: Innerhalb von 4 h werden > 80 % der Trinkmenge renal ausgeschieden. Dabei muss die Harnosmolalität < 100 mOsmol/kgH_2_O abfallen (Messung ca. 2 h nach Wasserbelastung).

Voraussetzungen: S‑Na^+^ > 125 mmol/l und Harnosmolalität > 100 mOsmol/kgH_2_O bei Testbeginn.

Messung der S‑Natriumkonzentration, der Harnosmolalität vor sowie danach stündlich bis Stunde 4 nach Testbeginn. Messung der Harnmenge.

SIAD: S‑Na^+^ sinkt unter den Ausgangswert, Harnosmolalität bleibt > 100 mOsmol/kgH_2_O.

Reset-Osmostat: S‑Na^+^ sinkt unter den Ausgangswert, Harnosmolalität sinkt < 100 mOsmol/kgH_2_O ab [245]. Die zu diesem Zeitpunkt gemessene S‑Natriumkonzentration entspricht in etwa der osmotischen Schwelle zur ADH-Sekretion.

## Abkürzungen

ADH: Antidiuretisches Hormon (Synonym für Vasopressin)
CVVHF: Kontinuierliche venovenöse Hämofiltration
EABV: Effektiv zirkulierendes arterielles Blutvolumen
EFWC: Elektrolytfreie Wasserclearance
EZV: Extrazellularvolumen
GFR: Glomeruläre Filtrationsrate
H‑Na^+^: Harn-Natriumkonzentration (mmol/l)
H‑UN: Harn-Urea-Nitrogen (Harnstoff-Stickstoff im Harn); im Vergleich zu Harnstoff-Stickstoff im Blut, „blood urea nitrogen“ [BUN])
kgKG: Kilogramm Körpergewicht
ODS: Osmotisches Demyelinisierungssyndrom
RAAS: Renin-Angiotensin-Aldosteron-System
SIAD: Syndrom der inadäquaten Antidiurese
SIADH: Syndrom der inadäquaten ADH-Sekretion
S‑Na^+^: Serum-Natrium (in mmol/l)
VExUS: Venous excess ultrasound (Score zur Abschätzung der venösen Kongestion)
