# Correction to: Konsensusempfehlungen zur Diagnose und Therapie der Hyponatriämie der Österreichischen Gesellschaft für Nephrologie 2024

**DOI:** 10.1007/s00508-024-02454-x

**Published:** 2024-10-07

**Authors:** Christoph Schwarz, Gregor Lindner, Martin Windpessl, Maarten Knechtelsdorfer, Marcus D. Saemann

**Affiliations:** 1 Innere Medizin 1, Pyhrn-Eisenwurzenklinikum, Sierningerstr. 170, 4400 Steyr, Österreich; 2 Zentrale Notaufnahme, Kepler Universitätsklinikum GmbH, Johannes-Kepler-Universität, Linz, Österreich; 3 Innere Medizin IV, Klinikum Wels-Grieskirchen, Wels, Österreich; 4 6.Medizinische Abteilung mit Nephrologie und Dialyse, Klinik Ottakring, Wien, Österreich; 5 Medizinische Fakultät, Sigmund-Freud Universität, Wien, Österreich

**Erratum:**

**Wien Klin Wochenschr 2024**

10.1007/s00508-024-02325-5

In diesem Beitrag ist die Formel 3 auf Seite 5 fehlerhaft.

Die korrekte Formel ist:

3 $$\begin{aligned}&\text{EFWC}\ (\mathrm{L})={}\\ &\text{Harnvolumen}\ (\mathrm{L})*\left(1-\frac{\text{H-Na+K}\ \left(\frac{\mathrm{mmol}}{\mathrm{L}}\right)}{\text{S-Na}\ \left(\frac{\mathrm{mmol}}{\mathrm{L}}\right)}\right)\end{aligned}$$

EFWC…: Elektrolyt freie Wasserclearance,
S‑Na…: Natriumkonzentration im Serum in mmol/L,
H‑Na+K…: Natrium- und Kaliumkonzentration im Harn in mmol/L.

Der Originalbeitrag wurde korrigiert.

